# Synthesis and Pharmacological Characterization of New Photocaged Agonists for Histamine H_3_ and H_4_ Receptors

**DOI:** 10.3390/ph17040536

**Published:** 2024-04-21

**Authors:** Yang Zheng, Meichun Gao, Maikel Wijtmans, Henry F. Vischer, Rob Leurs

**Affiliations:** Division of Medicinal Chemistry, Faculty of Science, Amsterdam Institute of Molecular and Life Sciences, Vrije Universiteit Amsterdam, 1081 HZ Amsterdam, The Netherlands; y.zheng@vu.nl (Y.Z.); m.c.gao@vu.nl (M.G.); m.wijtmans@vu.nl (M.W.); h.f.vischer@vu.nl (H.F.V.)

**Keywords:** photocaging, BODIPY cage, histamine, H_3_ receptor, H_4_ receptor

## Abstract

The modulation of biological processes with light-sensitive chemical probes promises precise temporal and spatial control. Yet, the design and synthesis of suitable probes is a challenge for medicinal chemists. This article introduces a photocaging strategy designed to modulate the pharmacology of histamine H_3_ receptors (H_3_R) and H_4_ receptors (H_4_R). Employing the photoremovable group BODIPY as the caging entity for two agonist scaffolds—immepip and 4-methylhistamine—for H_3_R and H_4_R, respectively, we synthesized two BODIPY-caged compounds, **5** (VUF25657) and **6** (VUF25678), demonstrating 10–100-fold reduction in affinity for their respective receptors. Notably, the caged H_3_R agonist, VUF25657, exhibits approximately a 100-fold reduction in functional activity. The photo-uncaging of VUF25657 at 560 nm resulted in the release of immepip, thereby restoring binding affinity and potency in functional assays. This approach presents a promising method to achieve optical control of H_3_R receptor pharmacology.

## 1. Introduction

Photopharmacology represents a new and cutting-edge area in the field of chemical biology, offering precise spatiotemporal control over biological processes [1,2,3,4]. Photopharmacology involves the use of light to modulate the activity of bioactive compounds, providing a non-invasive means to influence cellular functions with high precision [1,2,3,4]. Photocaging is one of the important strategies employed in the field of photopharmacology and this approach involves the temporary inactivation of a bioactive molecule through a light-sensitive protecting group [5,6,7,8].Upon exposure to light, the caging group is removed, releasing the bioactive molecule to act on its respective target. This approach holds great potential for studying and modulating cellular signaling pathways, potentially offering unprecedented spatial and temporal control of receptor activation.

In this study, we report our efforts in the field of photopharmacology of G protein-coupled receptors (GPCR) by developing photocaged agonists specifically tailored for histamine H_3_- and H_4_ receptors (H_3_R and H_4_R). The H_3_R and H_4_R are Gα_i_-coupled GPCRs and integral components of the histaminergic system [9,10]. Both GPCRs play a pivotal role in regulating various physiological processes and are emerging as promising targets for therapeutic intervention [9,10]. The H_3_R is primarily localized in the central nervous system, modulating the release of neurotransmitters such as histamine, dopamine, and acetylcholine. The intricate involvement of the H_3_R in cognitive functions, sleep-wake cycles, and appetite regulation underscores its significance as a potential target for neurological and psychiatric disorders [11,12,13]. Moreover, recently an H_3_R inverse agonist/antagonist (pitolisant) was approved for therapeutic use in narcolepsy (Wakix^®^) or in apnea patients suffering from excessive daytime sleepiness (Ozawade^®^) [14,15,16]. On the other hand, the H_4_R, although more recently discovered, has sparked considerable interest due to its expression in the immune system [9,10]. As an immune modulator, the H_4_R is implicated in the regulation of inflammatory responses, making it an attractive target for conditions involving immune dysregulation, such as allergies and autoimmune diseases [17,18]. Recently, its role in neurological disorders has also gained attention [19].

To allow spatio-temporal studies of the function of H_3_R and H_4_R, we focused on the design, synthesis, and characterization of novel photocaged agonists to enable spatiotemporal control of histamine receptor activity. Previously, caging groups such as nitrobenzyl, coumarin, and BODIPY (boron-dipyrromethene) have been successfully employed in the field of photocaging [2,6,7,8,20]. Especially, BODIPY dyes have gained interest as caging groups in view of their photostability and strong absorbance in the visible region [21]. This last property makes BODIPY compounds well-suited for light-triggered applications in biological context, as UV (ultraviolet) light is well-known to damage biomolecules. In this study, we report on the synthesis and characterization of BODIPY-caged immepip and 4-methylhistamine, two small molecule agonists known to act on H_3_R and H_4_R [22,23]. Caged compounds were analyzed for their photochemical properties, aggregation, and molecular pharmacological properties in radioligand binding and GPCR signaling studies.

## 2. Results

### 2.1. Design, Synthesis, and Photochemical Characterization of Photocaged Compounds

Following the discovery of immepip as a potent H_3_R agonist, various modifications have been reported in structure–activity relationship (SAR) studies. Among those, three earlier described modifications were instrumental in the design of photocaged immepip. First, the introduction of bulky N-alkyl substituents, such as an isopropyl group on the piperidine ring, by Kitbunnadaj et al. results in a remarkable >100-fold decrease in affinity [24]. Next, N-phenyl piperidine substituted immepip analogs prepared by Ishikawa et al. showed a pK_i_ range of 1–60 nM, representing 3 to 200-fold lower activity compared to immepip [25]. Lastly, the investigation by Vaccaro et al. of the basicity of the piperidine nitrogen revealed that analogs with a carbonyl group were 10 to >1500-fold less active towards H_3_R [26]. In contrast, so far very few immepip analogs with substituents on the imidazole N-atom have been reported [26]. Thus, in the design of our BODIPY-based photocaged H_3_R agonist, we considered photocaging of the piperidine amine functional group as the most viable approach.

In contrast to H_3_R agonists, there are significantly fewer reported H_4_R agonists, and many of them also exhibit activity towards H_3_R. In 2005, 4-methylhistamine was reported as a selective H_4_R agonist over H_3_R [22], making it an ideal candidate for the design of a photocaged H_4_R agonist. Within the structure of 4-methylhistamine, there are two potential positions for the introduction of the photocage group (the amino and imidazole groups). However, SARs of these two positions have not been well explored. We opted for attaching the required carbamate to the amino group rather than to the imidazole in view of its anticipated higher stability in the dark. Indeed, the recently published cryo-EM structures of histamine-bound H_4_R [27,28] indicate that the primary amino group of histamine direct or indirectly forms a salt bridge with Asp^943.32^ in transmembrane domain 3 and thus holds promise as a site for photocaging. From the three main classes of photocages in the literature, BODIPY was chosen in this study as photocaging group for the H_3_R/H_4_R ligands due to its lower-energy and rapid uncaging compared with o-nitrobenzyl and coumarin cages [29,30,31].

**Scheme 1:**
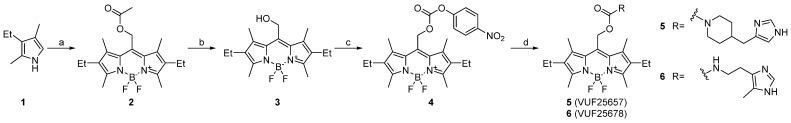
(**a**) (i) 2-chloro-2-oxoethyl acetate, DCM, reflux, 24 h; (ii) BF_3_·O(C_2_H_5_)_2_, TEA, rt, 30 min, 22%; (**b**) NaOH (aq.), MeOH, DCM, rt, 3 h, 42%; (**c**) 4-NO_2_PhOCOCl, pyridine, toluene, rt, 3 h, 87%; (**d**) R_1_NHR_2_, DIPEA, THF, rt, 4 h, 22% for **5** and 55% for **6**.

The synthesis of the BODIPY scaffold (Scheme 1) starts with pyrrole **1** [32,33,34,35]. By reacting this precursor with 2-chloro-2-oxoethyl acetate, followed by the addition of boron trifluoride diethyl etherate, **2** was obtained in a moderate yield. Subsequent hydrolysis provided alcohol **3**. By reacting alcohol **3** with 4-nitrophenyl chloroformate, key intermediate **4** was obtained in a high yield. BODIPY-caged analogs **5** (VUF25657) and **6** (VUF25678) were prepared from **4** by a substitution reaction with immepip and 4-methylhistamine, respectively.

**Figure 1:**
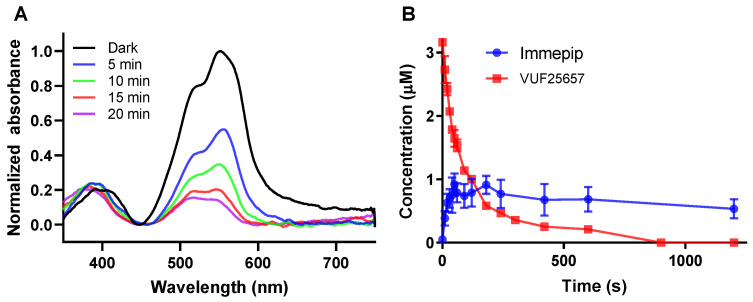
Photo-uncaging followed by UV–Vis (ultraviolet-visible) and LC–MS analysis. (**A**) VUF25657 (3.2 μM) was illuminated under 560 nm in Tris-HCl buffer (50 mM, pH 7.4)/1% DMSO at room temperature and a UV–Vis spectrum was measured at intervals. Only the 350–750 nm region is shown; (**B**) VUF25657 (3.2 μM) was illuminated under green LED light in Tris-HCl buffer (50 mM, pH 7.4)/1% DMSO at 37 °C and LC–MS analysis was performed at different time points using an internal standard.

Next, the photochemical properties of the caged VUF25657 and VUF25678 were investigated. Due to the large conjugation system of the BODIPY cage, the aqueous solubility of two caged compounds is limited to 3.2 μM, as determined by the nephelometry measurements (Figure S1). Based on the photochemical stability results in Figure S2, photo characterizations of VUF25657 and VUF25678 were performed under red LED light. Due to the low absorbance of immepip and 4-methylhistamine at 230 and 254 nm, a quantitative analysis method based on mass (MS) detection was employed. First, calibration curves of monitored compounds with the corresponding reference compound of the same molecular weight were built (Figure S3). Then, after illumination, the reference compounds were added to LCMS samples as internal standards. Based on the MS signal areas, the concentrations of caged compounds and parent compounds could be monitored. In Tris-HCl buffer (50 mM, pH 7.4), the absorption maximum (λ_max_) was determined to be 551 nm for VUF25657 (Figure 1A) and 546 nm for VUF25678 (Figure S4A). Within 20 min of continuous illumination of VUF25657 at 3.2 μM in Tris-HCl buffer with 560 ± 5 nm, the absorption centered around 550 nm decreased significantly (Figure 1A), and the active ligand immepip could be obtained (Figure 1B). The same results were observed for caged-4-methylhistamine (VUF25678) (Figure S4). After 180 s of illumination, the concentration of immepip reached 0.92 μM, amounting to 29% uncaging efficiency. Further illumination led to a decreased immepip concentration (for example, 0.53 μM at 20 min). One potential explanation for this could be the reaction of immepip with side products or intermediates formed during the uncaging process. A similar uncaging profile was observed for VUF25678 (Figure S4B).

### 2.2. Photopharmacological Characterization

**Figure 2:**
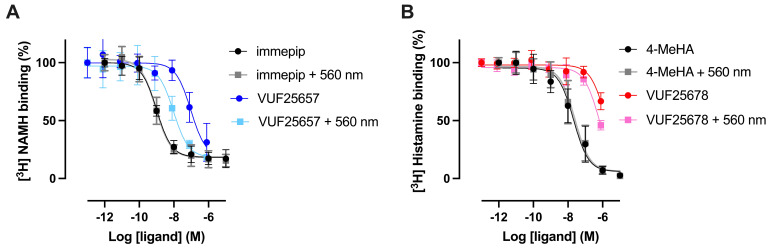
Displacement binding curves of parent, caged, and uncaged ligands for H_3_R (**A**) and H_4_R (**B**). Binding affinities were determined by displacement of the binding of 2 nM [^3^H]NAMH and 4 nM [^3^H]histamine to H_3_R and H_4_R, respectively. Caged ligands were either kept in dark or pre-irradiated at 560 nm for 3 min for VUF25657 and 5 min for VUF25678 to achieve the uncaged state. Data shown are mean ± S.D. from three or four independent experiments, each performed in triplicate.

The photocaged H_3_R and H_4_R agonists VUF25657 and VUF25678, either kept in the dark or pre-irradiated at 560 nm to achieve an uncaged state, were evaluated for their binding affinities by competition binding of the radioligands N-α-[methyl-^3^H] histamine ([^3^H]NAMH) and [^3^H]histamine for the human H_3_R and H_4_R, respectively. Both GPCRs were transiently expressed in HEK293T cells. Previously, we have shown that [^3^H]NAMH and [^3^H]histamine saturably bind to the H_3_R and H_4_R, respectively, with K_D_ values of 1.37 and 4.40 nM [36]. The parent compounds immepip and 4-methylhistamine (4-MeHA) potently displaced radioligand binding to H_3_R (Figure 2A) or H_4_R (Figure 2B). Moreover, pre-irradiation of the parent agonist solution at 560 nm did not lead to any difference in affinity (Figure 2A,B). The BODIPY-caged immepip (VUF25657) displayed a 125-fold decreased affinity for H_3_R. Illumination of VUF25657 at 560 nm partially restored the observed H_3_R affinity, with a 12-fold increase in comparison to its caged state (Table 1). Caging 4-methylhistamine with BODIPY also led to a decreased affinity for H_4_R. However, the displacement of radioligand binding by VUF25678 could not be measured at concentrations > 1 µM due to solubility issues. Therefore, the accurate affinity shift between caged and uncaged VUF25678 could not be determined and pharmacological analysis of VUF25678 was halted.

**Figure 3:**
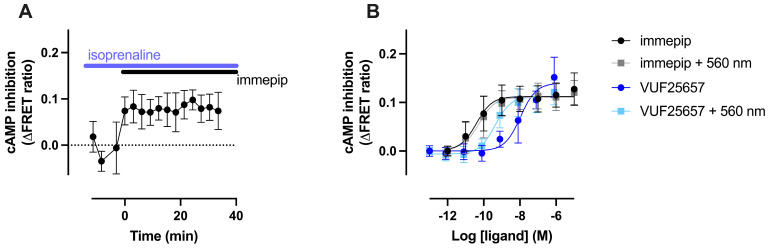
Optical modulation of VUF25657-induced cAMP inhibition in EPAC-H_3_R cells. Real time modulation of isoprenaline-induced cAMP acumination in HEK-EPAC cells by 1 μM immepip (**A**). Modulation of isoprenaline-induced cAMP acumination in HEK-EPAC cells by increasing concentrations of VUF25657 (**B**). HEK-EPAC cells stably expressing H_3_R were pre-stimulated with 10 nM isoprenaline for 10 min before ligand addition. The FRET signal of the EPAC-sensor was measured after 20 min treatment of indicated ligands. VUF25657 was either kept in dark or pre-irradiated at 560 nm for 3 min to release the BODIPY cage. Data are mean ± S.D. from four independent experiments performed, each in triplicate.

Next, the photocaged H_3_R agonist VUF25657, which notably displays a significant alteration in binding affinity to H_3_R upon irradiation at 560 nm, was further evaluated for its functionality by a FRET-based EPAC cAMP (cyclic adenosine monophosphate) biosensor [37]. H_3_R primarily couples to Gα_i_ protein, which upon activation causes a decrease in intracellular cAMP levels via the inhibition of adenylate cyclase. Therefore, the HEK-EPAC cells were pre-treated with 10 nM of the beta2 adrenergic receptor (β_2_AR) agonist isoprenaline before H_3_R stimulation to enhance the basal intracellular cAMP levels via β_2_AR-Gα_s_ activation. As anticipated, the H_3_R selective agonist immepip reduced the isoprenaline-induced cAMP accumulation, as detected by an increased FRET (fluorescence resonance energy transfer) signal in the EPAC (exchange protein directly activated by cAMP) biosensor (Figure 3A). In line with the binding affinity results, caging immepip with BODIPY (VUF25657) resulted in a decrease potency of cAMP inhibition (316-fold) as compared to immepip. Furthermore, as expected, irradiation of VUF25657 at 560 nm restored the activity for cAMP inhibition, demonstrated a significantly higher potency than its caged state (20-fold) (Figure 3B, Table 1).

## 3. Discussion

As an important component of photopharmacology, the photocaging strategy has a long history, compared to the photoswitch strategy, in controlling bioactivity by light. Dating back to the 1980s [38], the photocage strategy had already proven its important role in chemistry, biology, and other related fields [39]. To serve the purpose of spatiotemporal modulation of biological processes, several classes of photocages have been discovered and the concept has been well-developed so far [40]. In this study, the BODIPY cage was successfully coupled to selective H_3_R/H_4_R agonists and the resulting photocaged ligands could rapidly release the parent agonists. The novel photocaged H_3_R ligand VUF25657 was successfully applied in pharmacological assays. This new chemical biology tool exhibits significant activity differences in both H_3_R binding affinity and functional H_3_R assay before and after uncaging with visible light.

Despite the successful application, certain limitations of the BODIPY cage were also noted in this study. Compared to *o*-nitrobenzyl and coumarin cages, the BODIPY scaffold has a larger conjugation system, resulting in a lower-energy uncaging. However, this larger conjugation system often leads to compounds with low solubility in aqueous environments. The compound concentrations of VUF25657 and VUF25678 in both binding and functional assay had to be limited to maximally 3.2 µM, as determined by nephelometry. Due to this low concentration and the low absorbance of immepip and 4-methylhistamine at a common UV detection wavelength (such as 254 and 230 nm), a more complex MS-based analytical method than the routine LC–MS was employed to track the uncaging process. In our experiments with the caged H_4_R ligand, the release of the parent compound 4-methylhistamine could be monitored. However, due to the low concentration in the experiment and low activity of 4-methylhistamine, no significant difference was observed in the binding affinity assay before or after irradiation. To improve the aqueous solubility of BODIPY cages, Kand et al. have developed a water-soluble MESNA-BODIPY (2-mercaptoethanesulfonic acid-BODIPY) cage by introducing a sulfonic acid group [41]. However, the applicability of compounds with such a cage may be reduced depending on the target location, as deprotonation might impede the accessibility of their target. Nevertheless, for photocaging of GPCR ligands the future use of water-soluble BODIPY analogs might be a good way forward. 

Unexpectedly, the concentration of the desired parent compounds showed a decreasing trend after prolonged illumination. As proposed by Goswami et al. [32], uncaging of BODIPY analogs in aqueous solutions will yield a BODIPY alcohol next to the desired products. However, this desired alcohol was not easily observed in our uncaging process. Instead, a mass corresponding to a BODIPY-Tris byproduct was detected. We also hypothesized that the photo-bleaching products and/or reactive intermediates of the uncaging process could potentially further react with our desired uncaged compounds under these illumination conditions, thus leading to the decreased concentration of parent compounds.

In conclusion, in the present study we report on the successful BODIPY caging of the H_3_R/H_4_R agonists immepip and 4-methylhistamine and their evaluation with respect to photo-uncaging and subsequent target engagement. Our results indicate that VUF25657, the caged immepip, is the first successful, caged photopharmacological agent for spatio-temporal control of H_3_R function.

## 4. Materials and Methods

### 4.1. Chemistry

General information. The raw materials for our experiments were either purchased from commercial suppliers or available in our in-house inventory and used without additional purification. Solvents used in the procedures, such as THF, DCM, DMF, and toluene, were subjected to a purification process through an activated alumina column before use. Experimental procedures were conducted under a N_2_ environment unless mentioned otherwise. Thin-layer chromatography (TLC) analyses were employed using Merck F_254_ aluminum-backed silica plates and examined with a 254 nm UV lamp for compound detection. Separation and purification of reaction mixtures were achieved through flash column chromatography, utilizing the Biotage Isolera system. High resolution mass spectrometry analyses were performed on a Bruker microTOF mass spectrometer with electrospray ionization (ESI) in positive-ion mode. Nuclear magnetic resonance (NMR) spectroscopy was conducted on either a Bruker Avance 500 or 600 MHz instrument with a standard temperature (25 °C). The peak multiplicity patterns are categorized as follows: singlet (s), doublet (d), triplet (t), quartet (q), pentet (p), doublet of doublets (dd), doublet of triplets (dt), triplet of doublets (td), broad (br), and multiplet (m). The spectra were internally referenced to the NMR solvent peak at the following chemical shifts: CDCl_3_ at 7.26 ppm in ^1^H NMR and 77.16 ppm in ^13^C NMR; DMSO-*d*_6_ 2.50 ppm in ^1^H NMR and 39.52 ppm in ^13^C NMR; and CD_3_OD 3.35 ppm in ^1^H NMR and 49.00 ppm in ^13^C NMR. Then, 2D NMR HSQC and HMBC techniques were utilized to assign ^13^C signals if needed. IUPAC names were standardized using ChemBioDraw Ultra 19.0. The purity of the synthesized compounds was evaluated as the peak area percentage of the analyzed compound at 254 nm using liquid chromatography-mass spectrometry, which is equipped with a Shimadzu LC-20AD pump and a Shimadzu SPDM20A diode array detector. The mass spectrometry detection was facilitated with a Shimadzu LCMS-2010EV instrument, operating in positive ionization mode. The chromatographic separation was achieved on an Xbridge C18 column (5 μm, 100 mm × 4.6 mm). The following solutions were used as the eluents: solvent A: H_2_O with 0.1% HCOOH; and solvent B: MeCN with 0.1% HCOOH. A standard eluent method was used, unless mentioned otherwise: flow rate: 1.0 mL/min, 0–4.5 min, 95–10% A in a linear gradient; 4.5–6.0 min, 10% A; 6.0–6.5 min 10–95% A in a linear gradient; and 6.5–8.0 min 95% A. Final compounds (**5** and **6**) were >95% pure by HPLC analysis. 

#### 4.1.1. (2,8-Diethyl-5,5-difluoro-1,3,7,9-tetramethyl-5H-4λ^4^,5λ^4^-dipyrrolo [1,2-c:2′,1′-f][1,3,2]diazaborinin-10-yl)methyl acetate, **2**

To a solution of 3-ethyl-2,4-dimethyl-1*H*-pyrrole (14.5 mL, 0.107 mol, 2.0 eq) in DCM (642 mL) was added 2-chloro-2-oxoethyl acetate (5.8 mL, 53.7 mmol, 1.0 eq). The mixture was stirred under reflux for 24 h and then cooled to RT. TEA (44.8 mL, 0.332 mol, 6.0 eq) was added followed by the addition of BF_3_·O(C_2_H_5_)_2_ (59.7 mL, 0.484 mol, 9.0 eq) and the mixture was stirred at RT for 30 min. The solvent was reduced under vacuum and the dark oily residue was purified by silica gel chromatography with cyclohexane/DCM 30–70% to yield the title compound as a red solid (4.5 g, 22%). ^1^H NMR (600 MHz, CDCl_3_) δ 5.32 (s, 2H), 2.51 (s, 6H), 2.39 (q, *J* = 7.6 Hz, 4H), 2.25 (s, 6H), 2.14 (s, 3H), 1.05 (t, *J* = 7.6 Hz, 6H). ^13^C NMR (151 MHz, CDCl_3_) δ 170.8, 155.2, 136.7, 133.7, 132.4, 131.7, 58.5, 20.8, 17.3, 14.8, 12.8 (t, *J* = 2.8 Hz), 12.7. ^11^B NMR (160 MHz, CDCl_3_) δ 0.60 (t, *J* = 33.0 Hz). ^19^F NMR (471 MHz, CDCl_3_) δ −145.9 (q, *J* = 32.6 Hz). LC–MS: t_R_ = 5.59 min, purity: 93%, *m*/*z* [M + H]^+^: 377. Spectral data are in agreement with previous reports [32,33,34].

#### 4.1.2. (2,8-Diethyl-5,5-difluoro-1,3,7,9-tetramethyl-5H-4λ^4^,5λ^4^-dipyrrolo [1,2-c:2′,1′-f][1,3,2]diazaborinin-10-yl)methanol, **3**

A mixture of 0.10 M aq.NaOH solution (6.3 mL, 0.40 eq) and MeOH (30.0 mL) was stirred for 10 min and then added to a solution of **2** (550 mg, 1.46 mmol, 1.0 eq) in DCM (15.0 mL). The reaction mixture was stirred for 3 h in the dark at RT. The solvents were partially evaporated and the residue was extracted with EtOAc (3 × 30 mL). The combined organic layers were washed with 1 M HCl (2 × 20 mL), brine (20 mL), and dried over anhydrous MgSO_4_. The residue was purified by silica gel chromatography with cyclohexane/Et_2_O 30–100% to yield the title compound as a red solid (205 mg, 42%). ^1^H NMR (500 MHz, CDCl_3_) δ 4.93 (s, 2H), 2.50 (s, 6H), 2.44–2.37 (m, 10H), 1.05 (t, *J* = 7.6 Hz, 6H). ^13^C NMR (151 MHz, CDCl_3_) δ 154.7, 136.7, 136.5, 133.5, 131.8, 56.3, 17.3, 14.9, 12.78, 12.76. ^11^B NMR (160 MHz, CDCl_3_) δ 0.60 (t, *J* = 33.3 Hz). ^19^F NMR (471 MHz, CDCl_3_) δ −146.0 (q, *J* = 33.0 Hz). LC–MS: t_R_ = 5.13 min, purity: >99%, *m*/*z* [M + H]^+^: 335. Spectral data are in agreement with previous reports [33,34,35].

#### 4.1.3. (2,8-Diethyl-5,5-difluoro-1,3,7,9-tetramethyl-5H-4λ^4^,5λ^4^-dipyrrolo [1,2-c:2′,1′-f][1,3,2]diazaborinin-10-yl)methyl (4-nitrophenyl) carbonate, **4**

To a stirred solution of **3** (1.62 g, 4.83 mmol, 1.0 eq) in PhMe (132 mL) at RT, 4-nitrophenyl chloroformate (3.90 g, 19.3 mmol, 4.0 eq) and pyridine (1.9 mL, 24.2 mmol, 5.0 eq) were added. The reaction mixture was stirred at RT for 3 h. The reaction mixture was washed with satd. aq. NH_4_Cl (150 mL) and brine (150 mL). The organic layer was dried over MgSO_4_ and solvents were removed under reduced pressure. The residue was purified by gradient reverse phase column with MeCN/H_2_O with 0.1% HCOOH from 5–100% to yield the title compound as a red solid (2.10 g, 87%). ^1^H NMR (500 MHz, CDCl_3_) δ 8.32–8.27 (m, 2H), 7.44–7.37 (m, 2H), 5.59 (s, 3H), 2.53 (s, 6H), 2.41 (q, *J* = 7.6 Hz, 6H), 2.36 (s, 8H), 1.06 (t, *J* = 7.6 Hz, 6H). ^13^C NMR (126 MHz, CDCl_3_) δ 155.9, 155.4, 152.4, 145.7, 136.6, 134.1, 132.3, 129.3, 125.5, 121.8, 62.3, 17.3, 14.8, 13.0, 12.9. ^11^B NMR (160 MHz, CDCl_3_) δ 0.59 (t, *J* = 32.9 Hz). ^19^F NMR (471 MHz, CDCl_3_) δ −143.1 (q, *J* = 32.9 Hz). LC–MS: t_R_ = 5.86 min, purity: 96.6%, *m*/*z* [M + H]^+^: 500. Spectral data are in agreement with a previous report [35].

#### 4.1.4. (2,8-Diethyl-5,5-difluoro-1,3,7,9-tetramethyl-5*H*-4λ^4^,5λ^4^-dipyrrolo [1,2-*c*:2′,1′-*f*][1,3,2]diazaborinin-10-yl)methyl 4-((1*H*-imidazol-4-yl)methyl)piperidine-1-carboxylate, **5**

To a stirred solution of **4** (0.15 g, 0.30 mmol, 1.0 eq) in THF (4.5 mL) at RT was added 4-((1*H*-imidazol-4-yl)methyl)piperidine·2HBr (0.15 g, 0.45 mmol, 1.5 eq) and DIPEA (0.26 mL, 1.5 mmol, 5.0 eq). The reaction mixture was stirred at RT for 4 h. Upon completion, the reaction mixture was diluted with EtOAc (8.0 mL). The organic phase was washed with satd. aq. NH_4_Cl (6.0 mL) and brine (6.0 mL), dried over MgSO_4_, and filtered. Solvents were removed under reduced pressure. The crude product was purified by silica gel chromatography (0–2% MeOH in DCM) to yield the title compound as a red solid (35 mg, 22%). ^1^H NMR (600 MHz, DMSO-*d*_6_) δ 11.89–11.62 (m, 1H), 7.47 (s, 1H), 6.82–6.52 (m, 1H), 5.25 (s, 2H), 4.06–3.73 (m, 2H), 2.85–2.70 (m, 2H), 2.46–2.33 (m, 12H), 2.26 (s, 6H), 1.77–1.49 (m, 3H), 1.00 (m, 8H). ^13^C NMR (151 MHz, CDCl_3_) δ 154.9, 154.8, 154.8, 143.8, 137.6, 136.9, 133.9, 133.6, 132.5, 132.4, 116.1, 115.1, 59.0, 44.4, 44.2, 36.0, 35.6, 34.9, 32.6, 32.1, 31.7, 29.8, 17.3, 14.9, 12.8, 12.7, 12.7. Extra peaks were observed, likely as a result of rotamers.^11^B NMR (160 MHz, CDCl_3_) δ 0.69 (t, *J* = 32.3 Hz). ^19^F NMR (471 MHz, CDCl_3_) δ −145.8 (q, *J* = 30.4 Hz). LC–MS: t_R_ = 4.02 min, purity: >99%, *m*/*z* [M + H]^+^: 526. HR-MS: calcd for C_28_H_38_BF_2_N_5_O_2_ [M + H]^+^, 526.3159; found, 526.3185. m.p., 148.7–150.7 °C.

#### 4.1.5. (2,8-Diethyl-5,5-difluoro-1,3,7,9-tetramethyl-5*H*-4λ^4^,5λ^4^-dipyrrolo [1,2-*c*:2′,1′-*f*][1,3,2]diazaborinin-10-yl)methyl (2-(5-methyl-1*H*-imidazol-4-yl)ethyl)carbamate, **6**

To a stirred solution of **4** (0.15 g, 0.30 mmol, 1.0 eq) in dry THF (5.0 mL) at RT was added 2-(5-methyl-1*H*-imidazol-4-yl)ethan-1-amine·2HCl (89 mg, 0.45 mmol, 1.5 eq) and DIPEA (0.26 mL, 1.5 mmol, 5.0 eq). The reaction mixture was divided into ten aliquots and stirred at that temperature for 4 h. Upon completion, all aliquots were combined and diluted with EtOAc (10.0 mL). The organic phase was washed with satd. aq. NH_4_Cl (6.0 mL) and brine (6.0 mL), dried with MgSO_4_ and filtered. Solvents were removed under reduced pressure. The crude product was purified by silica gel chromatography (0–1% MeOH in DCM) to yield the title compound as a red solid (95 mg, 55%). ^1^H NMR (600 MHz, CD_3_OD) δ 7.51 (s, 1H), 5.30 (s, 2H), 3.33 (d, *J* = 7.0 Hz, 2H), 2.76–2.66 (m, 2H), 2.49–2.41 (m, 10H), 2.32 (s, 6H), 2.14 (s, 3H), 1.06 (t, *J* = 7.6 Hz, 6H). ^13^C NMR (151 MHz, CDCl_3_) δ 158.2, 155.7, 138.4, 134.7, 134.3, 134.2, 133.5, 130.1, 127.5, 59.3, 41.9, 26.9, 17.9, 15.1 12.8, 12.7, 10.1. ^11^B NMR (160 MHz, CDCl_3_) δ 0.54 (t, *J* = 32.3 Hz). ^19^F NMR (471 MHz, CDCl_3_) δ −146.5 (q, *J* = 32.5 Hz). LC–MS: t_R_ = 3.70 min, purity: 96.8%, *m*/*z* [M + H]^+^: 486. HR-MS: calcd for C_25_H_34_BClF_2_N_4_O_2_ [M + H]^+^, 486.2846; found, 486.2870. m.p., 108.8–116.3 °C.

### 4.2. Nephelometry

Compounds under investigation were placed into clear, flat-bottom 96-well plates in the absence of light, each containing a distinct concentration (from 10^−4^ M to 10^−7.5^ M, with a blank as a baseline reference), and were left to stabilize in a phosphate buffer solution with 1% DMSO for a minimum of 60 min before measurement. A kaolin suspension was placed in each plate at varying concentrations (10^−4^ M to 10^−7.5^ M), and was subjected to identical experimental conditions as the test compounds [42]. Utilizing a NEPHELO star Plus instrument from BMG Labtech, Germany, turbidity measurements were taken under a set of parameters: fours cycles of readings, initiating the measurement at 0.1 s, with each subsequent reading at 0.1 s interval time. The laser intensity was set to 80%, the beam focus was adjusted to 2.0 mm, and the plates were agitated using an orbital shaking mode at 200 rpm, with an additional 10 s of shaking before the commencement of each cycle. Data analysis was carried out using GraphPad Prism 8 software with the collected data points, generating a line chart that displays both the mean and standard deviation values. These results were compared with the kaolin control to assess the tested compounds.

### 4.3. (Photo)chemical Stability Assay

Compound aqueous stability and room light stability tests (Figure S2) were carried out with 10 µM samples in 50 mM phosphate buffer plus 1% DMSO at room temperature in clear glass vials. Aqueous stability was checked hourly using LC detection at 254 nm, while room light stability was monitored every 5 min for an hour with identical LC settings.

### 4.4. Materials for Pharmacological Evaluation

Fetal bovine serum (FBS, #16170078) was obtained from Gibco (Thermo Fisher Scientific, Waltham, MA, USA). Penicillin/streptomycin (P/S) was purchased from GE Healthcare (Uppsala, Sweden). Dulbecco’s Modified Eagles Medium (DMEM, #41966-029), Dulbecco’s phosphate-buffered saline (DPBS, #15326239), 0.05% Trypsin-EDTA (#11580626), and Hanks’ Balanced Salt Solution (HBSS, #11560456) were bought from Thermo Fisher Scientific (Waltham, MA, USA). Linear poly-ethylenimine (PEI, 25-kDa, # 23966-1) was obtained from Polysciences (Warrington, PA, USA). G418 (#108321-42-2) and isoprenaline (#I6504, (*R*)-3,4-Dihydroxy-α-(isopropylaminomethyl)benzyl alcohol hydrochloride) were purchased from Sigma-Aldrich (St. Louis, MO, USA). Zeocin (#ZEL-43-05) was purchase from InvivoGen (San Diego, CA, USA). Black 96-well plates (#655086) were purchased from Greiner Bio-One (Frickenhausen, Germany). *N*^α^-[methyl-^3^H]histamine (#NET1027250UC), [^3^H]histamine (#NET732250UC), Microscint-O scintillation liquid (#6013611), GF/C filter plates (#6055690) and MicrobetaWallac Trilux scintillation counter were purchased from PerkinElmer (Groningen, The Netherlands).

### 4.5. Cell Culture and Transfection

HEK293T cells (ATTC, CRL-1573) were cultured in DMEM supplemented with 10% FBS and 1% P/S in a humidified incubator at 37 °C with 5% CO_2_. The HEK293 cell line stably expressing an EPAC-cAMP FRET biosensor was kindly provided by Dr. M. Zimmermann (Interax Biotech, Basal, Switzerland) [43,44]. HEK293-EPAC cells stably co-expressing the H_3_R (GenBank accession number NM_007232.3) were generated as previously described [37].

### 4.6. Membrane Preparation

Membranes were collected from HEK293T cells transiently transfected with H_3_R or H_4_R by PEI method. In brief, 2 million HEK293T cells were seeded into a 10 cm dish one day before transfection. The next day, the cells were transfected with 2.5 μg cDNA encoding H_3_R (GenBank accession number AF140538) or H_4_R (GenBank accession number AY136745) supplemented with 2.5 μg empty pcDEF3 [45] plasmid using 20 μg PEI. Two days after transfection, the transfected HEK293T cells were detached using ice-cold PBS, and the membrane pellets were subsequently collected by centrifuging at 1932× *g* at 4 °C for 10 min.

### 4.7. Radioligand Binding Assay

For competition binding assays, the tested compounds were pre-irradiated to reach the uncaged state. In brief, 0.3 mM stock of photocaged ligand (dissolved in DMSO) was divided into two samples, one of which was subsequently diluted in Tris-HCl buffer (50 mM, pH 7.4) to 3 μM and then irradiated by 560 nm to reach the uncaged state, as confirmed by LC–MS, and the other sample was kept in dark to retain its caged state. The prepared membrane pellets expressing H_3_R or H_4_R were resuspended in Tris-HCl buffer (50 mM, pH 7.4) and disrupted by 5 s sonication to make membrane suspension. Next, all the following handling was performed under near-infrared light. For testing the binding to H_3_R, membrane suspension expressing H_3_R was incubated with 2 nM N-α-[methyl-^3^H] histamine and increasing concentrations of unlabeled ligands prepared in Tris-HCl buffer. For testing the binding to H_4_R, binding assays were performed by displacing 4 nM [^3^H] histamine with increasing concentrations of unlabeled ligands prepared in Tris-HCl buffer on membrane suspension expressing H_4_R. Following one hour of incubation with continuous shaking at 225 rpm at 25 °C, the reaction was terminated by transferring the mixture to GF/C filter plates that were pre-soaked with a solution containing 0.5% PEI and washed with cold Tris-HCl buffer. After drying for 30 min at 55 °C, the radioactivity remaining on the filters was quantified using a Microbeta Wallac Trilux scintillation counter, following the addition of scintillation liquid with a two-hour delay.

### 4.8. cAMP Inhibition by FRET-EPAC Biosensor

EPAC-H_3_R cells were seeded into black 96-well plates with 50,000 cells per well one day before the experiment. The next day, the culture medium was replaced with HBSS. Subsequently, 10 nM isoprenaline (dissolved in HBSS supplemented with 20 mM thiourea) was added for 10 min incubation to elevate basal cAMP levels. Fluorescence resonance energy transfer (FRET) measurements were recorded by CLARIOstar plate reader in real-time or at 20 min after ligand stimulation.

### 4.9. Data Analysis

All data are shown as mean ± S.D. from three individual experiments performed in triplicate. Figures were generated and data analyzed by Prism 9. In radioligand binding assays, the competition binding curves were fitted by ‘one-site—Fit K_i_’ to obtain the equilibrium dissociation constants of unlabeled ligands (K_i_) by Cheng-Prusoff equation [46]. In FRET-EPAC assays, the ligand-induced cAMP inhibition was quantified by FRET ratios, dividing the FRET signal at 530 nm by the signal at 480 nm. The response was represented as ΔFRET ratios, obtained by the analysis of ‘Fractional difference: (Value—Baseline)/Baseline’. Then the concentration–response curves were fitted using the model: ‘log(agonist) vs. response (three parameters)’ to obtain potency (pEC_50_).

## 5. Conclusions

In conclusion, a BODIPY-based photocaging strategy appears a successful strategy to optically modulate histamine H_3_ receptor function. Photocaging of the H_3_R agonist immepip leads to **5** (VUF25657), which shows >100-fold lower affinity and potency than immepip. Photo-uncaging of **5** with 560 nm illumination led to the desired parent compound immepip, restoring its binding affinity and potency. These findings prove that **5** is a powerful new photoresponsive tool to modulate H_3_R pharmacology, offering promising avenues for future exploration in GPCR photopharmacology efforts.

## Figures and Tables

**Table 1:** Pharmacological data of photocaged H_3_R and H_4_R agonist. Statistical difference (*p* < 0.05) of pK_i_ and pEC_50_ values for the pre-irradiated compound in comparison to that in dark state was determined by unpaired *t*-test with Welch’s and indicated with an asterisk (*). Data are mean ± S.D. from three or four independent experiments performed in triplicate. N.D. = not determined.

Compound	pK_i_		pEC_50_	
	In Dark	+560 nm	In Dark	+560 nm
VUF25657 (**5**)	7.3 ± 0.6 (4)	8.4 ± 0.2 (4) *	8.0 ± 0.4 (4)	9.3 ± 0.2 (4) *
immepip	9.4 ± 0.2 (4)	9.4 ± 0.3 (4)	10.5 ± 0.6 (4)	10.2 ± 0.6 (4)
VUF25678 (**6**)	<6 (3)	<6 (3)	N.D.	N.D.
4-methylhistamine	7.2 ± 0.2 (3)	7.2 ± 0.1 (3)	N.D.	N.D.

## Data Availability

The original contributions presented in the study are included in the article/Supplementary Material, further inquiries can be directed to the corresponding author.

## Supplementary Materials

The following supporting information can be downloaded at: https://www.mdpi.com/article/10.3390/ph17040536/s1, Figure S1. Representative plots for nephelometry measurements at different concentrations of parent compounds (immepip and 4-methylhistamine) and caged-compounds **5** and **6** in the dark; Figure S2. Chemical stability of 5 (A/B/C) and 6 (D/E/F) under dark, red light and ambient light; Figure S3. MS calibration curves of the immepip, 4-methylhistamine and BODIPY-caged compounds (**5** and **6**) with their corresponding reference compounds; Figure S4. Photo-uncaging followed by UV–Vis and LC–MS analysis; Figures S5–S31. LC–MS, NMR and HRMS spectroscopy data.
