# Supplementary material for: Synthesis and Pharmacological Characterization of New Photocaged Agonists for Histamine H3 and H4 Receptors
Source: Pharmaceuticals (Basel). 2024 Apr 21;17(4):536. doi: 10.3390/ph17040536 (PMC11053687; doi:10.3390/ph17040536)
Supplement: Supplementary file 1 [file pharmaceuticals-17-00536-s001.zip › pharmaceuticals-2956618-supplementary.pdf]

Supporting information

Synthesis and pharmacological characterization of new photo-caged agonist for histamine H_3 and H_4 receptors

Yang Zheng[#], Meichun Gao[#], Maikel Wijtmans, Henry F. Vischer and Rob Leurs*

Division of Medicinal Chemistry, Faculty of Science, Amsterdam Institute of Molecular and Life Sciences, Vrije Universiteit Amsterdam, 1081 BV Amsterdam, The Netherlands.

[#] Both authors contributed equally.

* Correspondence: r.leurs@vu.nl

Table of contents

Figure S1.	Representative plots for nephelometry measurements at different concentrations of parent compounds (immepip and 4-methylhistamine) and caged-compounds VUF25657 and VUF25678 in the dark.	Page S2
Figure S2.	Chemical stability of VUF25657 (A/B/C) and VUF25678 (D/E/F) under dark, red light and ambient light.	Page S2
Figure S3.	MS calibration curves of the immepip, 4-methylhistamine and BODIPY-caged compounds (VUF25657 and VUF25657) with their corresponding reference compounds.	Page S3
Figure S4.	Photo-uncaging followed by UV-Vis and LC-MS analysis.	Page S4
Figures S5-S31.	LC-MS, NMR and HRMS spectroscopy data.	Page S5-22

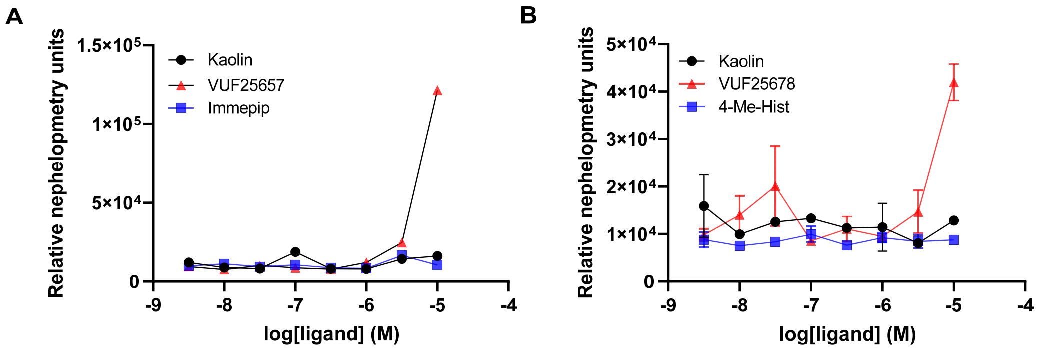

Figure S1. Representative plots for nephelometry measurements at different concentrations of parent compounds (immepip and 4-methylhistamine) and caged-compounds VUF25657 and VUF25678 in the dark. A kaolin dispersion was used as a positive control. Experiments were performed in duplicate in 50 mM Tris-HCl buffer (pH 7.4)/1 % DMSO.

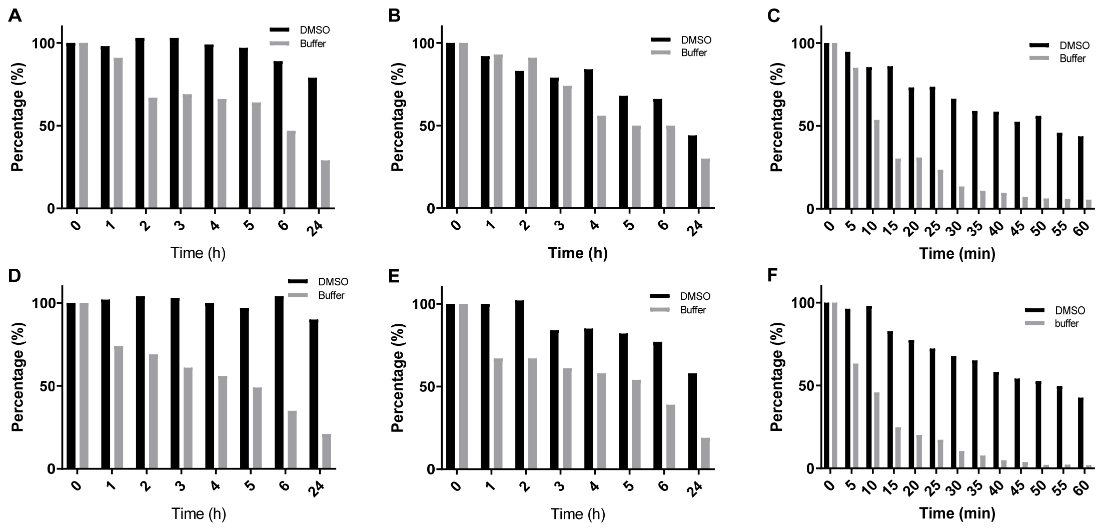

Figure S2. Chemical stability of VUF25657 (A/B/C) and VUF25678 (D/E/F) under dark, red light and ambient light. VUF25657 or VUF25678 (3.2 µM) was incubated in Tris-HCl buffer (50 mM, pH 7.4)/1% DMSO at room temperature and analysis by LC-MS (λ = 254 nm, area % of BODIPY-caged compounds versus internal standard (1,3,5-trimethoxylbenzene) was performed at different time points. Black columns: VUF25657 or VUF25678 in DMSO; Grey columns: VUF25657 or VUF25678 in Tris-HCl buffer (50 mM, pH 7.4)/1% DMSO.

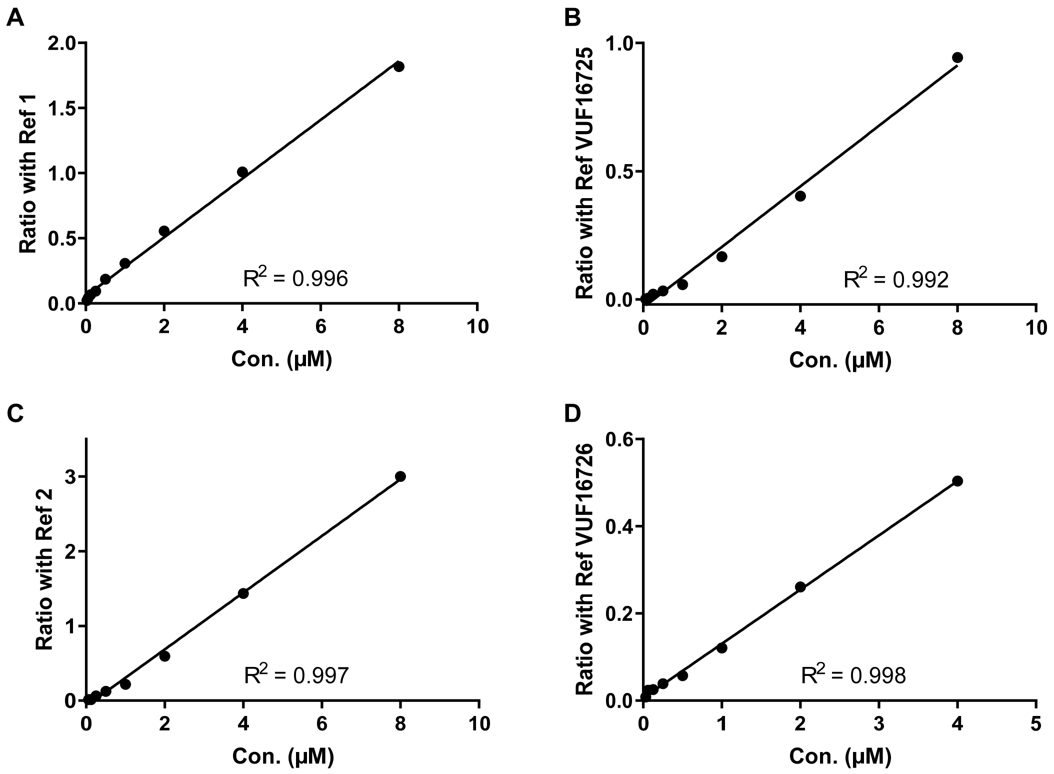

Figure S3. MS calibration curves of immepip, 4-methylhistamine and BODIPY-caged compounds VUF25657 and VUF25678 with their corresponding reference compounds. The ratio of peak areas is plotted. A, calibration curve of immepip with 3-dimethylaminobenzoic acid from Sigma-Aldrich (CAS: 99-64-9); B, calibration curve of VUF25657 with VUF16725 (from our in-house library); C, calibration curve of 4-methylhistamine with (4-fluorophenyl)methanamine Sigma-Aldrich (CAS: 140-75-0); D, calibration curve of VUF25658 with VUF16726 (from our in-house library).

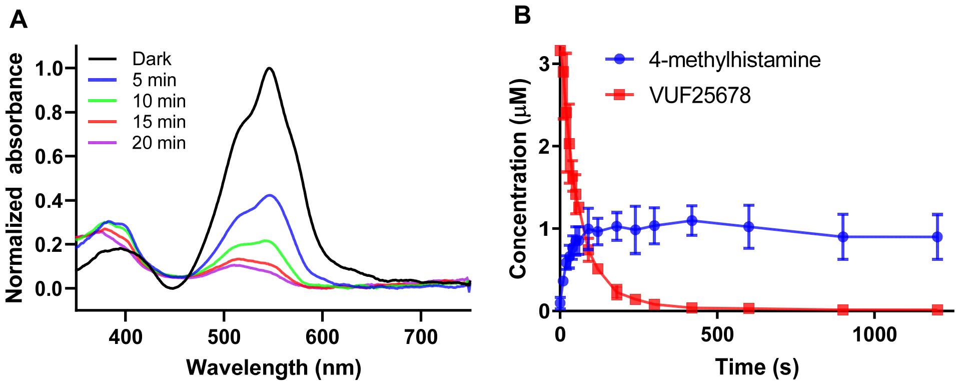

Figure S4. Photo-uncaging followed by UV-Vis and LC-MS analysis. (A) VUF25678 (3.2 μM) was illuminated under 560 nm in Tris-HCl buffer (50 mM, pH 7.4)/1% DMSO at room temperature and a UV-Vis spectrum measured at intervals; (B) VUF25678 (3.2 μM) was illuminated under green LED light in Tris-HCl buffer (50 mM, pH 7.4)/1% DMSO at 37 ℃ and LC-MS analysis performed at different time points.

Acquired by	: Admin
Date Acquired	: 18/8/2020 10:23:12 AM
Sample Name	: YAZH_122
Sample ID	:
Tray#	: 1
Vial#	: 4
Injection Volume	: 1
Data File	: C:\LabSolutions\Data\2020\2020-w34\YAZH_122.lcd
Background File	: azo blanco 20200818.lcd
Method File	: Method SCAN ACID standard azo.lcm
Report Format	: DefaultLCMS.lcr
Tuning File	: C:\LabSolutions\Tuning\Tuning-ESI-pos-neg01072015a.lct
Processed by	: Admin
Modified Date	: 18/8/2020 10:53:52 AM

PDA Graph

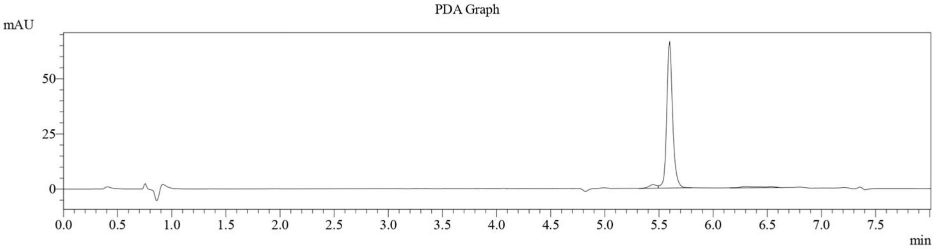

PeakTable

PDA Ch1 254nm 4nm

Peak#	Name	Ret. Time	Area	Area %
1		5.439	8554	3.195
2		5.592	249100	93.042
3		6.299	10075	3.763

MS Chromatogram
YAZH_122 C:\LabSolutions\Data\2020\2020-w34\YAZH_122.lcd

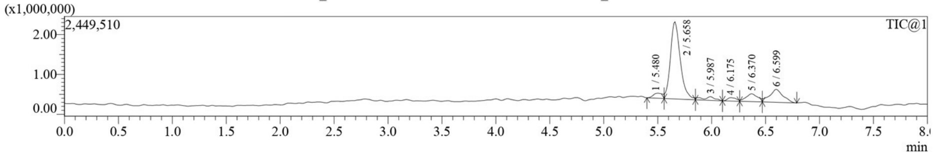

MS Spectrum Graph

#:1 Ret.Time:Averaged 5.650-5.670(Scan#:566-568)
BG Mode:Calc 5.560<->5.850(557<->586)
Mass Peaks:69 Base Peak:357.20(384449) Polarity:Pos Segment1 - Event1

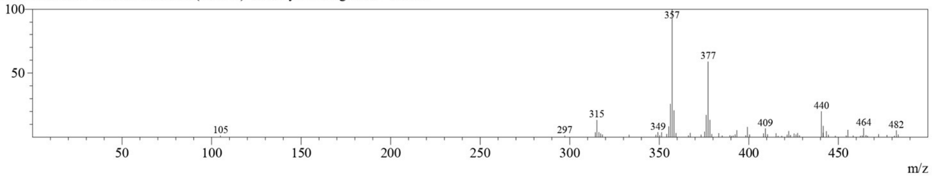

MS Spectrum Table

#:1 Ret.Time:
BG Mode:Calc 5.560<->5.850(557<->586)
Mass Peaks:69 Base Peak:357.20(384449) Polarity:Pos Segment1 - Event1

#	m/z	Abs.Inten.	Rel.Inten.	Charge	Polarity	Monoisotopic	#	m/z	Abs.Inten.	Rel.Inten.	Charge	Polarity	Monoisotopic
1	104.95	4498	1.17				10	349.20	15142	3.94			
2	297.25	4923	1.28				11	350.25	5921	1.54			
3	314.30	14792	3.85				12	351.30	14934	3.88			
4	315.20	51583	13.42				13	354.15	9459	2.46			
5	316.25	15112	3.93				14	355.30	32639	8.49			
6	317.25	11344	2.95				15	356.20	99960	26.00			
7	318.30	8087	2.10				16	357.20	384449	100.00			
8	333.15	7317	1.90				17	358.25	80507	20.94			
9	348.15	7420	1.93				18	359.35	12656	3.29			
19	366.30	5649	1.47				45	423.30	6657	1.73			
20	367.30	12989	3.38				46	425.30	11057	2.88			
21	373.35	7694	2.00				47	426.30	8104	2.11			
22	375.30	16803	4.37				48	427.25	12709	3.31			
23	376.20	66869	17.39				49	428.30	5596	1.46			
24	377.25	226563	58.93				50	440.50	77599	20.18			
25	378.25	52045	13.54				51	441.50	13354	3.47			

Figure S5. LCMS chromatogram of intermediate **2**.

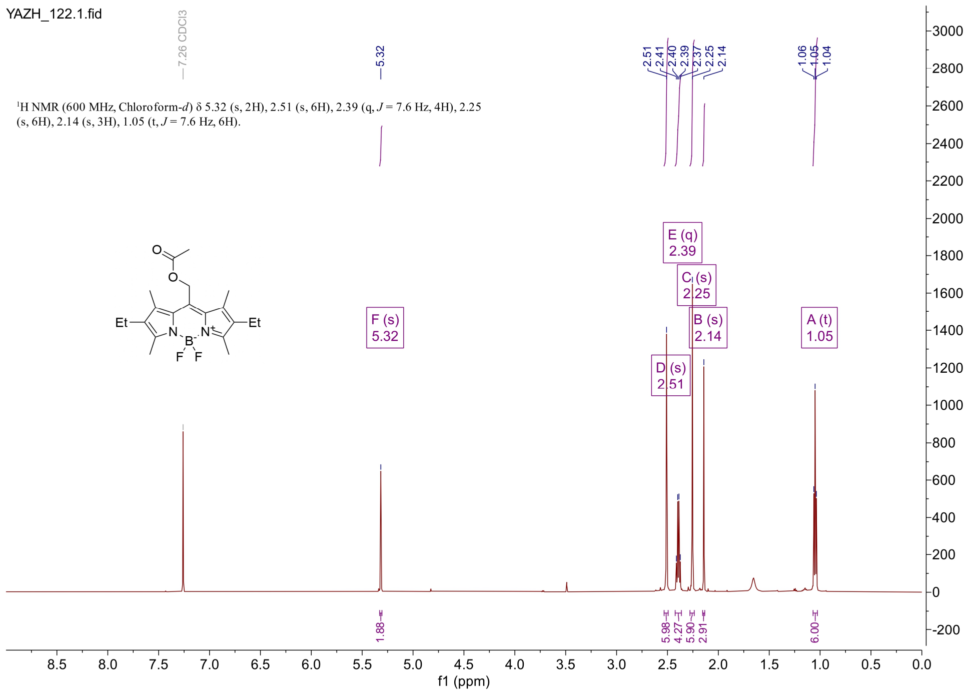

Figure S6. ¹H NMR spectrum of intermediate **2**.

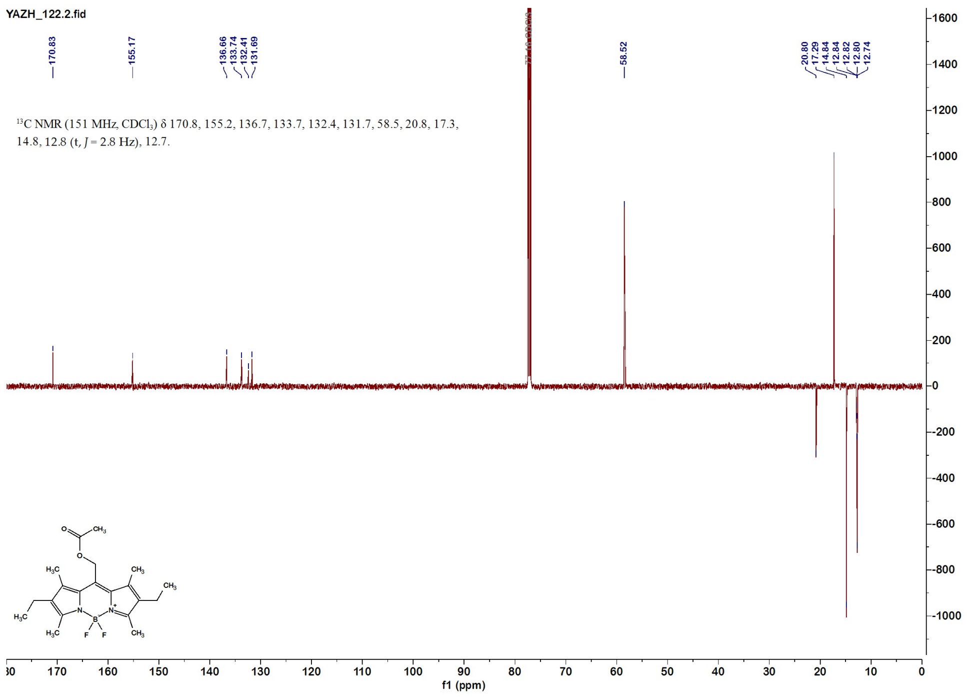

Figure S7. ¹³C NMR spectrum of intermediate **2**.

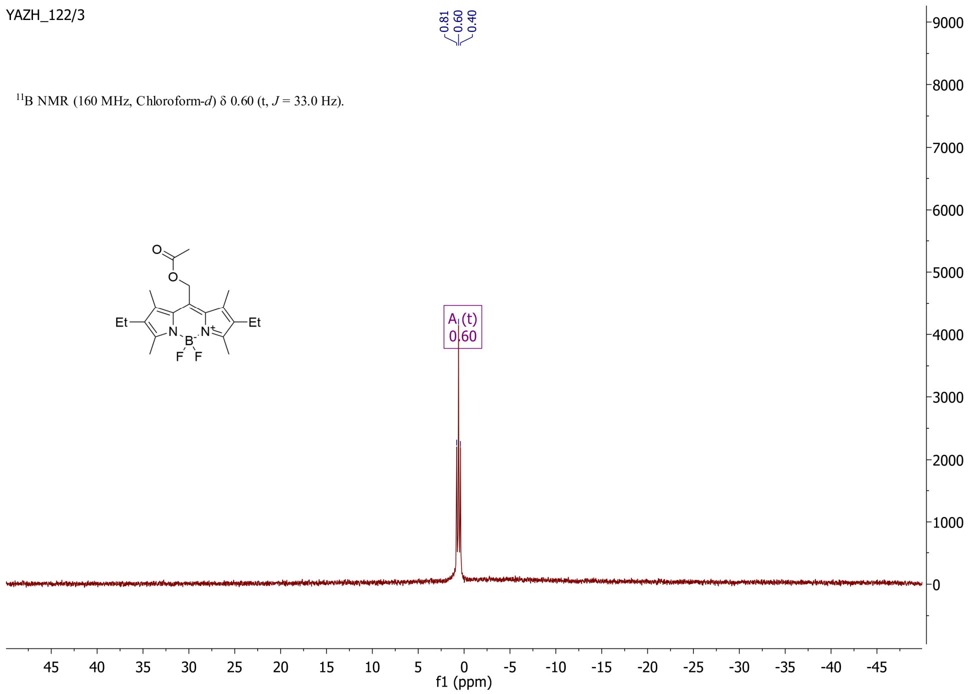

Figure S8. ^{11}B NMR spectrum of intermediate **2**.

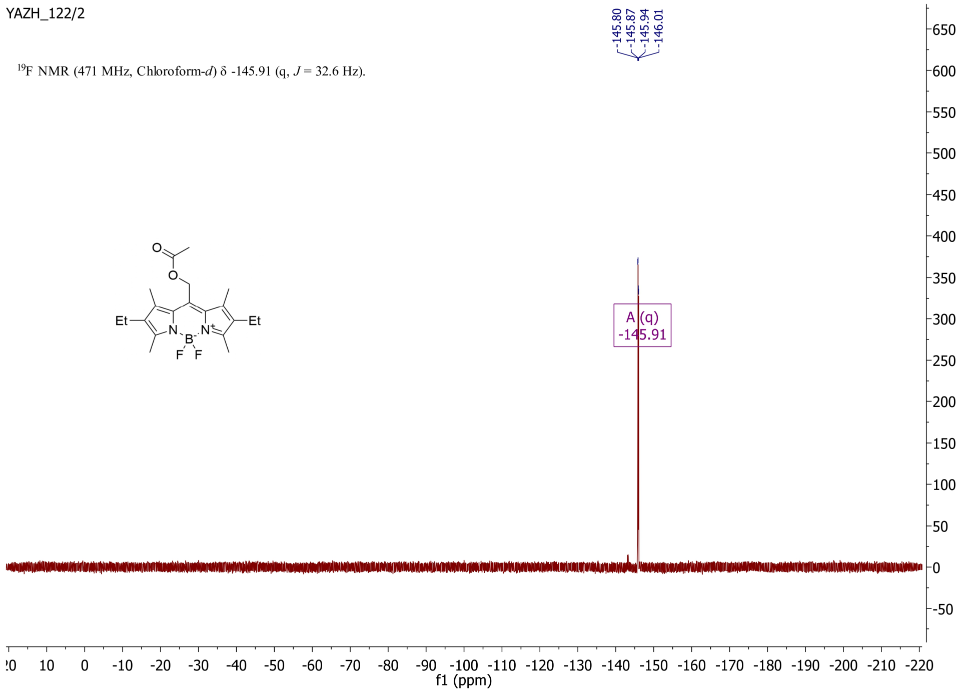

Figure S9. ^{19}F NMR spectrum of intermediate **2**.

Acquired by	: Admin
Date Acquired	: 24/8/2020 10:13:56 AM
Sample Name	: YAZH_123_20200824
Sample ID	:
Tray#	: 1
Vial#	: 2
Injection Volume	: 5
Data File	: C:\LabSolutions\Data\2020\2020-w34\YAZH_123_20200824.lcd
Background File	: azo blanco 24082020.lcd
Method File	: Method SCAN ACID standard azo.lcm
Report Format	: DefaultLCMS.lcr
Tuning File	: C:\LabSolutions\Tuning\Tuning-ESI-pos-neg01072015a.lct
Processed by	: Admin
Modified Date	: 24/8/2020 10:31:04 AM

PDA Graph

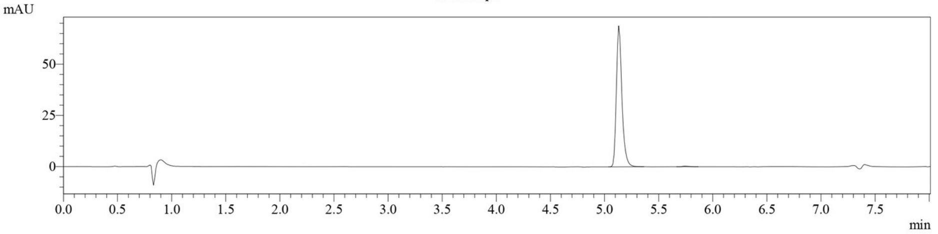

PeakTable

PDA Ch1 254nm 4nm

Peak#	Name	Ret. Time	Area	Area %
1		5.127	247298	99.395
2		5.739	1504	0.605

MS Chromatogram

YAZH_123_20200824 C:\LabSolutions\Data\2020\2020-w34\YAZH_123_20200824.lcd

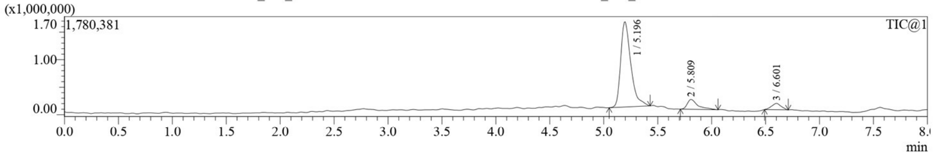

MS Spectrum Graph

#:1 Ret.Time:Averaged 5.190-5.210(Scan#:520-522)
BG Mode:Calc 5.050<->5.430(506<->544)
Mass Peaks:55 Base Peak:315.20(368490) Polarity:Pos Segment1 - Event1

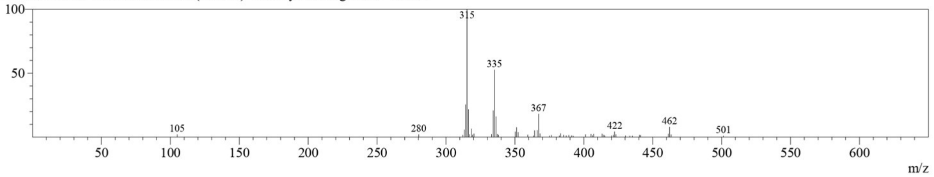

MS Spectrum Table

#:1 Ret.Time:
BG Mode:Calc 5.050<->5.430(506<->544)
Mass Peaks:55 Base Peak:315.20(368490) Polarity:Pos Segment1 - Event1

#	m/z	Abs.Inten.	Rel.Inten.	Charge	Polarity	Monoisotopic
1	105.00	8069	2.19			
2	280.25	8193	2.22			
3	312.30	6651	1.80			
4	313.30	21923	5.95			
5	314.30	94237	25.57			
6	315.20	368490	100.00			
7	316.20	79927	21.69			
8	317.25	8518	2.31			

#	m/z	Abs.Inten.	Rel.Inten.	Charge	Polarity	Monoisotopic
9	318.35	24544	6.66			
10	319.15	6928	1.88			
11	320.25	10001	2.71			
12	333.10	8590	2.33			
13	334.30	76925	20.88			
14	335.20	194192	52.70			
15	336.30	59729	16.21			
16	337.35	8941	2.43			

Figure S10. LCMS chromatogram of intermediate **3**.

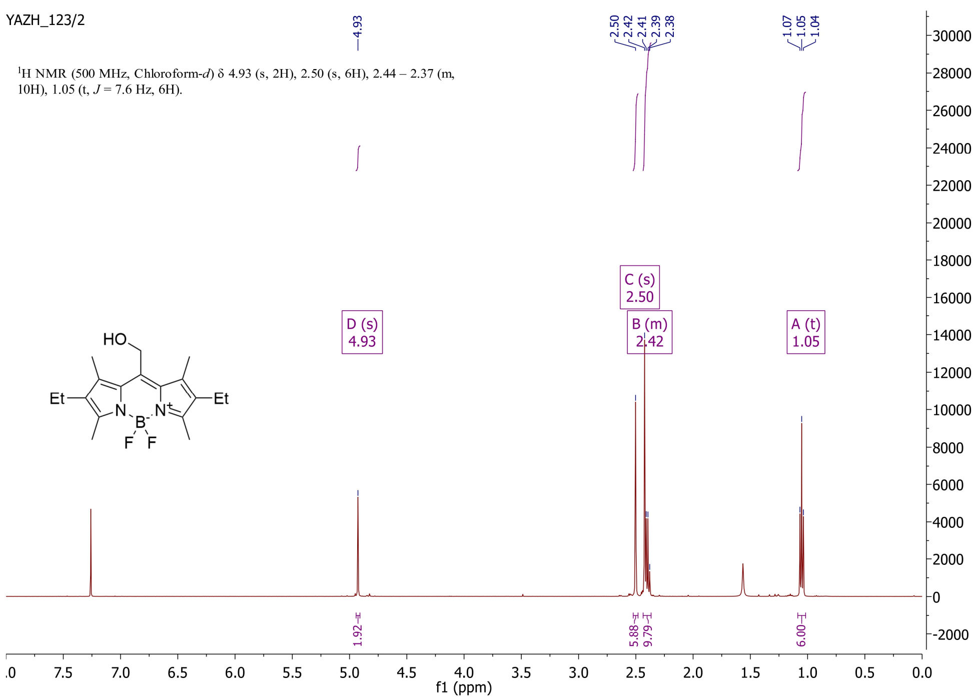

Figure S11. ^{1}H NMR spectrum of intermediate **3**.

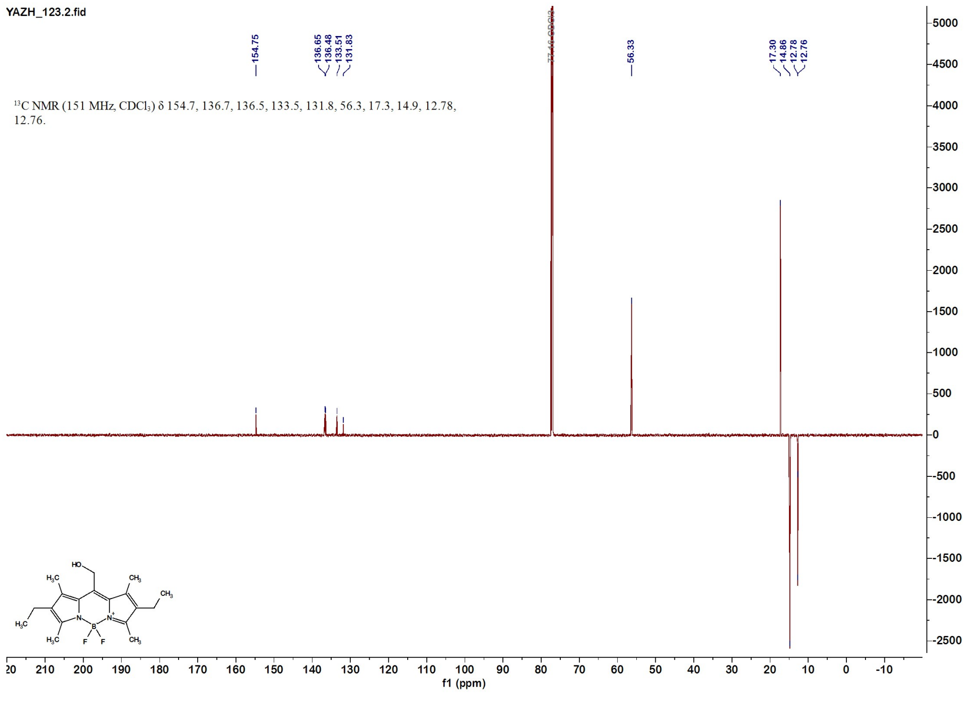

Figure S12. ^{13}C NMR spectrum of intermediate **3**.

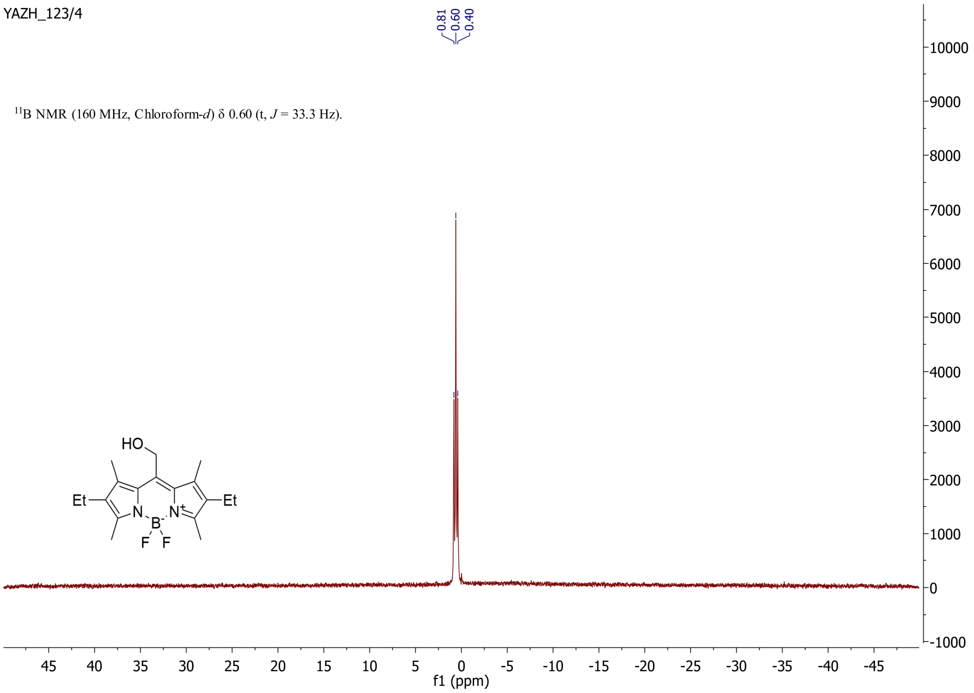

Figure S13. ^{11}B NMR spectrum of intermediate **3**.

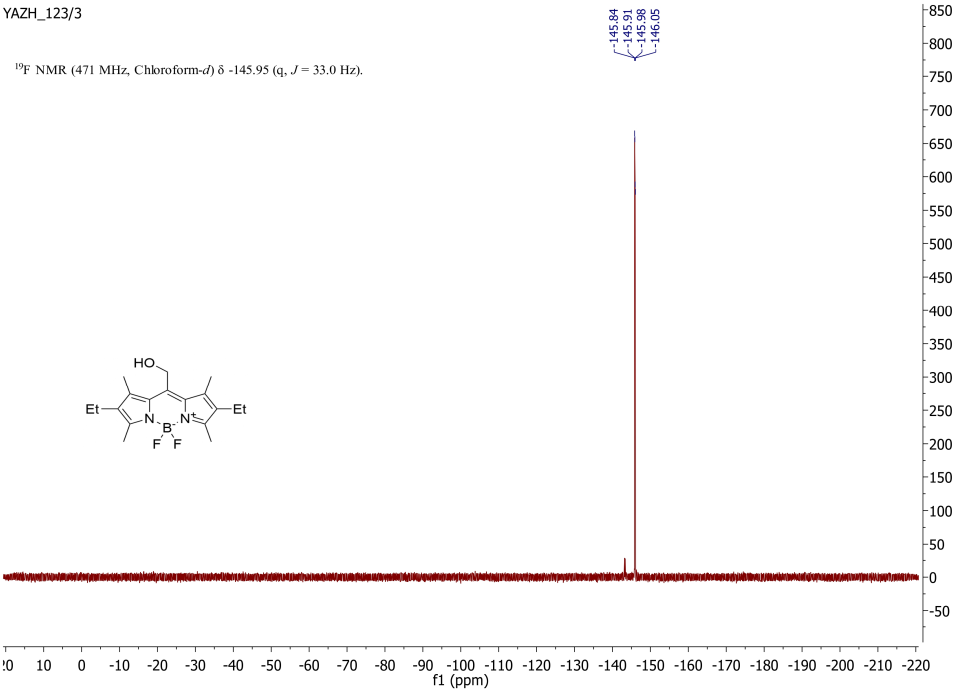

Figure S14. ^{19}F NMR spectrum of intermediate **3**.

Acquired by	: Admin
Date Acquired	: 18/1/2021 10:20:44 AM
Sample Name	: YAZH_149
Sample ID	:
Tray#	: 1
Vial#	: 5
Injection Volume	: 1
Data File	: C:\LabSolutions\Data\2021\2021-wk03\YAZH_149.lcd
Background File	: azo blanco 18012021.lcd
Method File	: Method SCAN ACID standard Yang azo.lcm
Report Format	: DefaultLCMS.lcr
Tuning File	: C:\LabSolutions\Tuning\Tuning-ESI-pos-neg01072015a.lct
Processed by	: Admin
Modified Date	: 18/1/2021 10:54:38 AM

PDA Graph

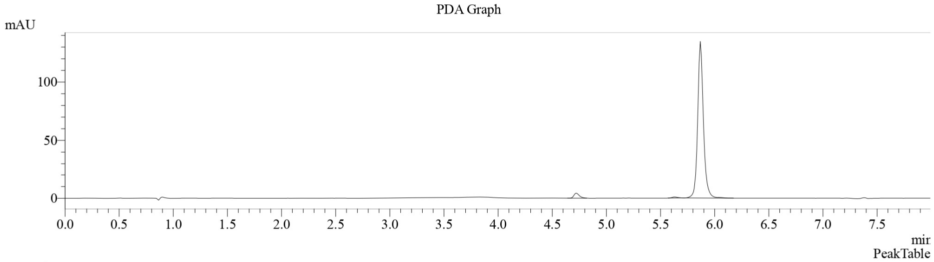

PeakTable

PDA Ch1 254nm 4nm

Peak#	Name	Ret. Time	Area	Area %
1		4.716	14839	2.854
2		5.625	2993	0.576
3		5.862	502115	96.571

MS Chromatogram
YAZH_149 C:\LabSolutions\Data\2021\2021-wk03\YAZH_149.lcd

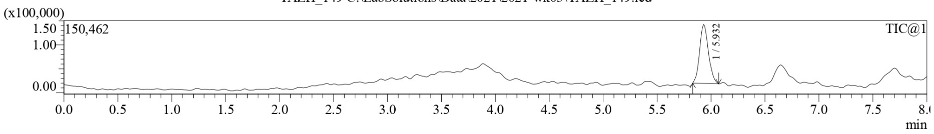

MS Spectrum Graph

#:1 Ret.Time:Averaged 5.920-5.940(Scan#:593-595)
BG Mode:Calc 5.830<->6.070(584<->608)
Mass Peaks:70 Base Peak:587.30(13909) Polarity:Pos Segment1 - Event1

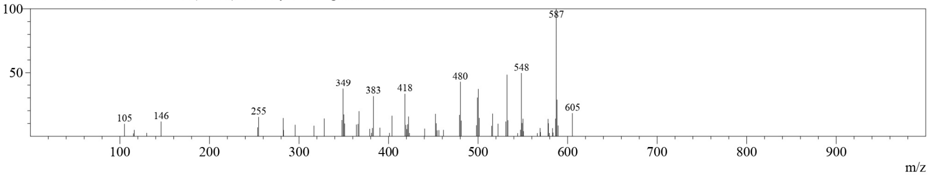

MS Spectrum Table

#:1 Ret.Time:
BG Mode:Calc 5.830<->6.070(584<->608)
Mass Peaks:70 Base Peak:587.30(13909) Polarity:Pos Segment1 - Event1

#	m/z	Abs.Inten.	Rel.Inten.	Charge	Polarity	Monoisotopic
1	105.00	1355	9.74			
2	115.00	315	2.26			
3	116.00	692	4.98			
4	129.95	364	2.62			
5	145.95	1602	11.52			
6	253.80	979	7.04			
7	254.80	2100	15.10			
8	282.30	1985	14.27			
9	282.75	689	4.95			

#	m/z	Abs.Inten.	Rel.Inten.	Charge	Polarity	Monoisotopic
10	295.75	1249	8.98			
11	316.70	1156	8.31			
12	328.15	1936	13.92			
13	348.05	1766	12.70			
14	349.10	5183	37.26			
15	350.05	2376	17.08			
16	351.00	1395	10.03			
17	364.05	1274	9.16			
18	365.95	1334	9.59			

#	m/z	Abs.Inten.	Rel.Inten.	Charge	Polarity	Monoisotopic
19	367.10	2727	19.61			
20	379.00	810	5.82			
21	380.95	369	2.65			
22	381.95	898	6.46			
23	383.15	4365	31.38			
24	390.35	930	6.69			
25	401.10	365	2.62			
26	403.90	2237	16.08			
27	418.35	4610	33.14			
28	419.40	1235	8.88			
29	420.35	789	5.67			
30	421.40	1307	9.40			
31	422.25	2152	15.47			
32	423.30	359	2.58			
33	440.35	829	5.96			
34	452.45	2446	17.59			
35	453.30	1412	10.15			
36	454.30	640	4.60			
37	456.35	670	4.82			
38	461.35	695	5.00			
39	479.25	2322	16.69			
40	480.30	5931	42.64			
41	481.20	1707	12.27			
42	498.20	1206	8.67			
43	499.20	4212	30.28			
44	500.30	5141	36.96			

#	m/z	Abs.Inten.	Rel.Inten.	Charge	Polarity	Monoisotopic
45	501.30	2004	14.41			
46	515.15	1135	8.16			
47	516.20	2468	17.74			
48	522.25	1365	9.81			
49	531.05	1604	11.53			
50	532.35	6752	48.54			
51	533.30	1746	12.55			
52	544.20	340	2.44			
53	547.35	714	5.13			
54	548.35	6934	49.85			
55	549.20	1446	10.40			
56	550.25	1906	13.70			
57	550.65	556	4.00			
58	566.15	338	2.43			
59	569.25	904	6.50			
60	569.65	460	3.31			
61	578.20	1882	13.53			
62	578.60	1421	10.22			
63	579.60	349	2.51			
64	583.20	880	6.33			
65	583.60	409	2.94			
66	586.45	1922	13.82			
67	587.30	13909	100.00			
68	588.20	3984	28.64			
69	589.30	1180	8.48			
70	605.30	2514	18.07			

Figure S15. LCMS chromatogram of intermediate **4**.

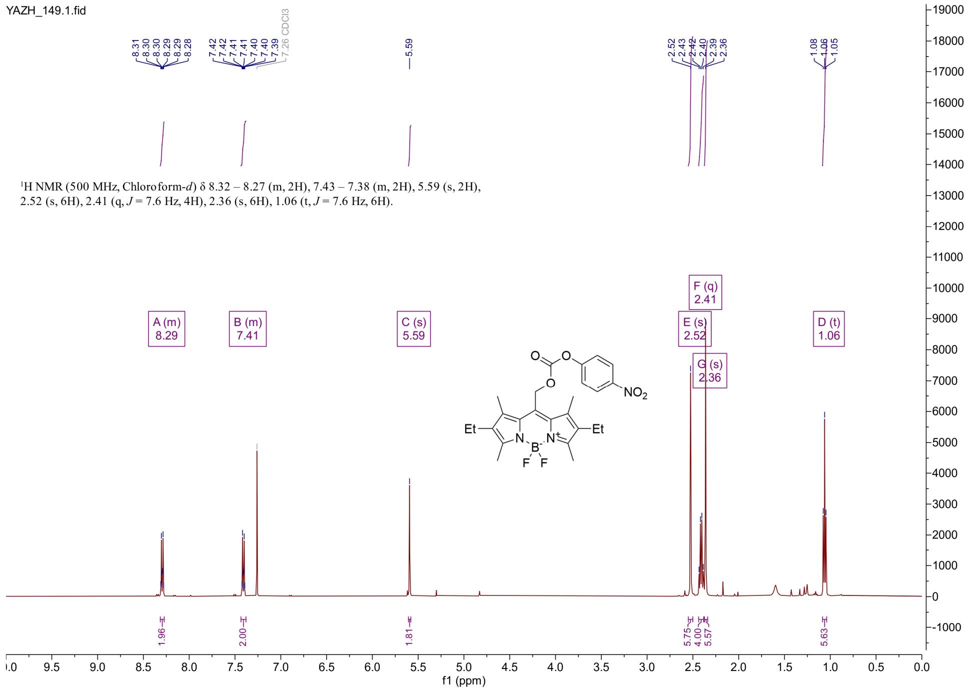

Figure S16. ^{1}H NMR spectrum of intermediate **4**.

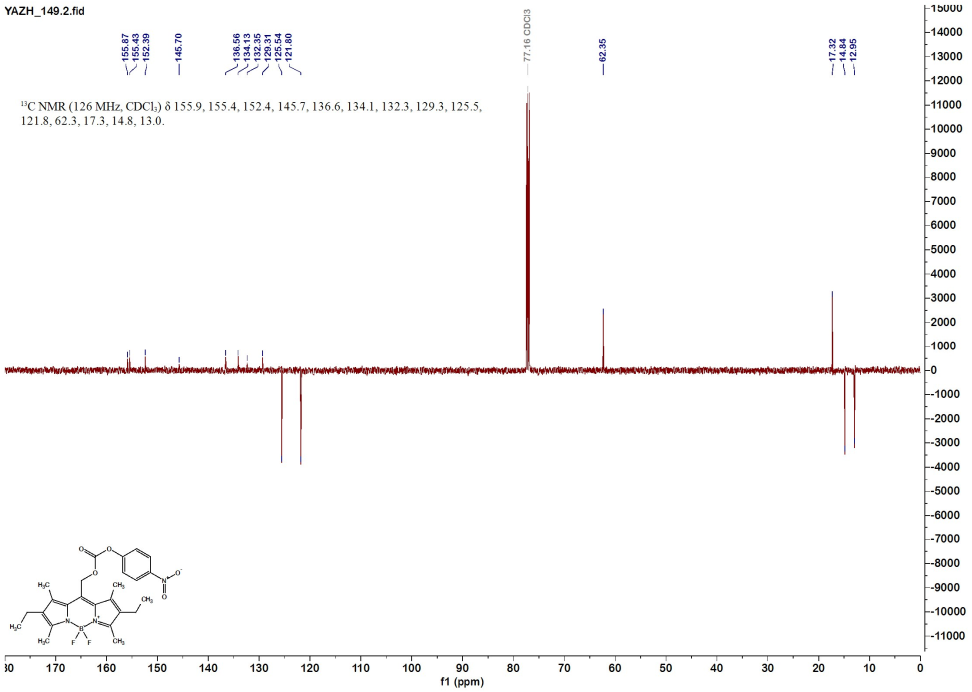

Figure S17. ^{13}C NMR spectrum of intermediate **4**.

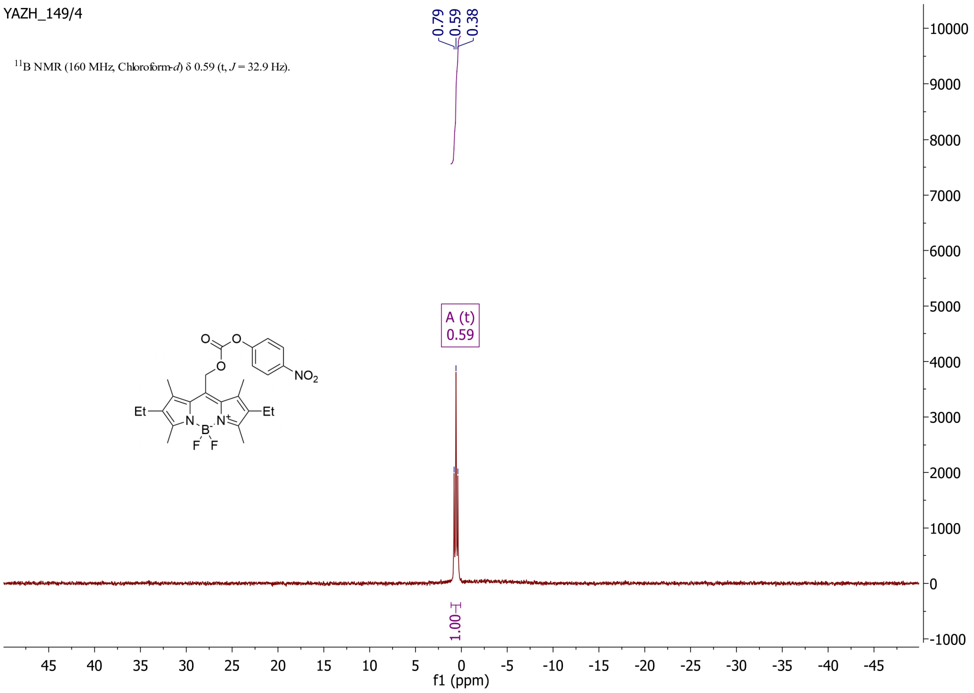

Figure S18. ^{11}B NMR spectrum of intermediate **4**.

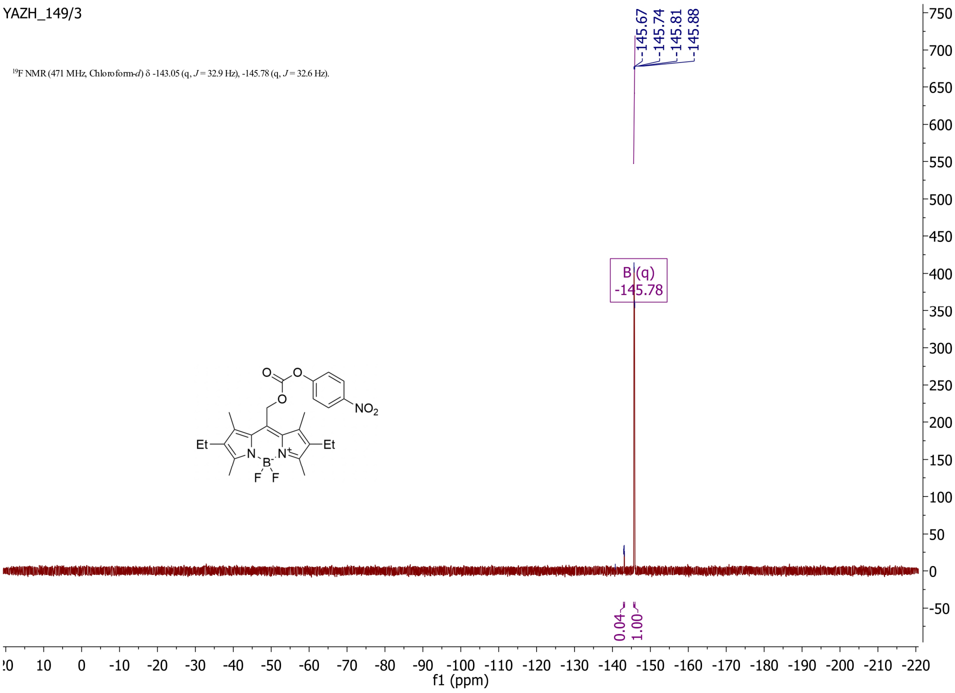

Figure S19. ^{19}F NMR spectrum of intermediate **4**.

Acquired by	: Admin
Date Acquired	: 28/5/2021 11:28:53 AM
Sample Name	: YAZH_176 20210528
Sample ID	:
Tray#	: 1
Vial#	: 16
Injection Volume	: 10
Data File	: C:\LabSolutions\Data\2021\2021-wk21\YAZH_176 20210528.lcd
Background File	: blanco 28052021.lcd
Method File	: Method SCAN ACID standard.lcm
Report Format	: DefaultLCMS.lcr
Tuning File	: C:\LabSolutions\Tuning\tuning 15042021.lct
Processed by	: Admin
Modified Date	: 28/5/2021 11:46:49 AM

PDA Graph

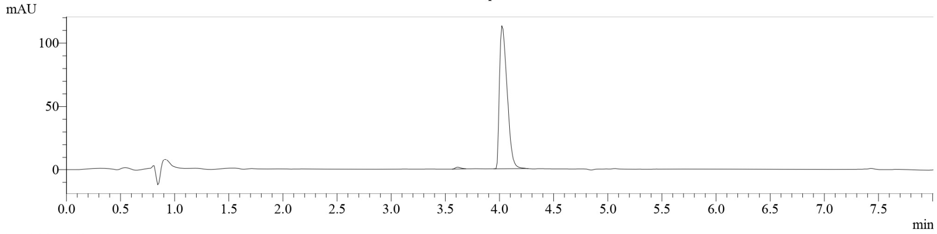

PDA Ch1 254nm 4nm

Peak#	Name	Ret. Time	Area	Area %
1		3.607	5041	0.894
2		4.019	558949	99.106

MS Chromatogram
YAZH_176 20210528 C:\LabSolutions\Data\2021\2021-wk21\YAZH_176 20210528.lcd

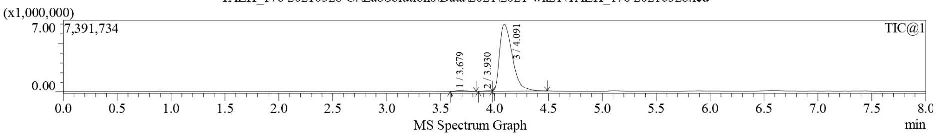

MS Spectrum Graph

#:1 Ret.Time:Averaged 4.080-4.100(Scan#:409-411)
BG Mode:Calc 3.980<->4.490(399<->450)
Mass Peaks:15 Base Peak:506.35(2165914) Polarity:Pos Segment1 - Event1

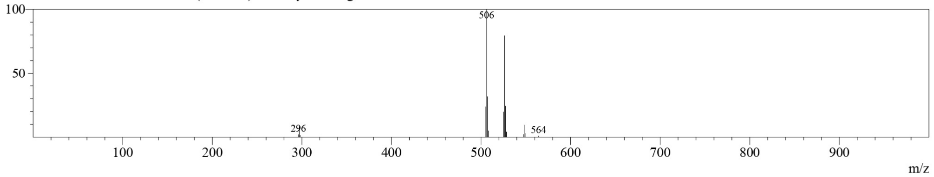

MS Spectrum Table

#:1 Ret.Time:
BG Mode:Calc 3.980<->4.490(399<->450)
Mass Peaks:15 Base Peak:506.35(2165914) Polarity:Pos Segment1 - Event1

#	m/z	Abs.Inten.	Rel.Inten.	Charge	Polarity	Monoisotopic
1	296.20	48018	2.22			
2	297.10	182775	8.44			
3	298.10	35108	1.62			
4	505.30	516176	23.83			
5	506.35	2165914	100.00			
6	507.30	686504	31.70			
7	508.35	111055	5.13			
8	525.35	428810	19.80			

#	m/z	Abs.Inten.	Rel.Inten.	Charge	Polarity	Monoisotopic
9	526.35	1725061	79.65			
10	527.35	528346	24.39			
11	528.35	91724	4.23			
12	547.30	50778	2.34			
13	548.30	208443	9.62			
14	549.35	67822	3.13			
15	564.30	24446	1.13			

Figure S20. LCMS chromatogram of final compound 5.

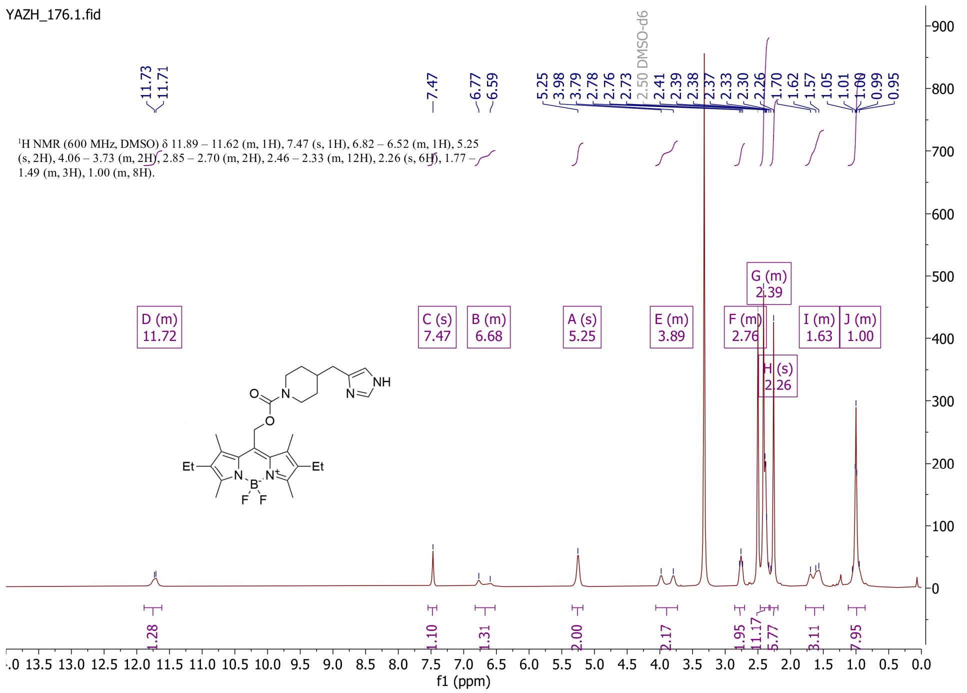

Figure S21. ^{1}H NMR spectrum of final compound **5**.

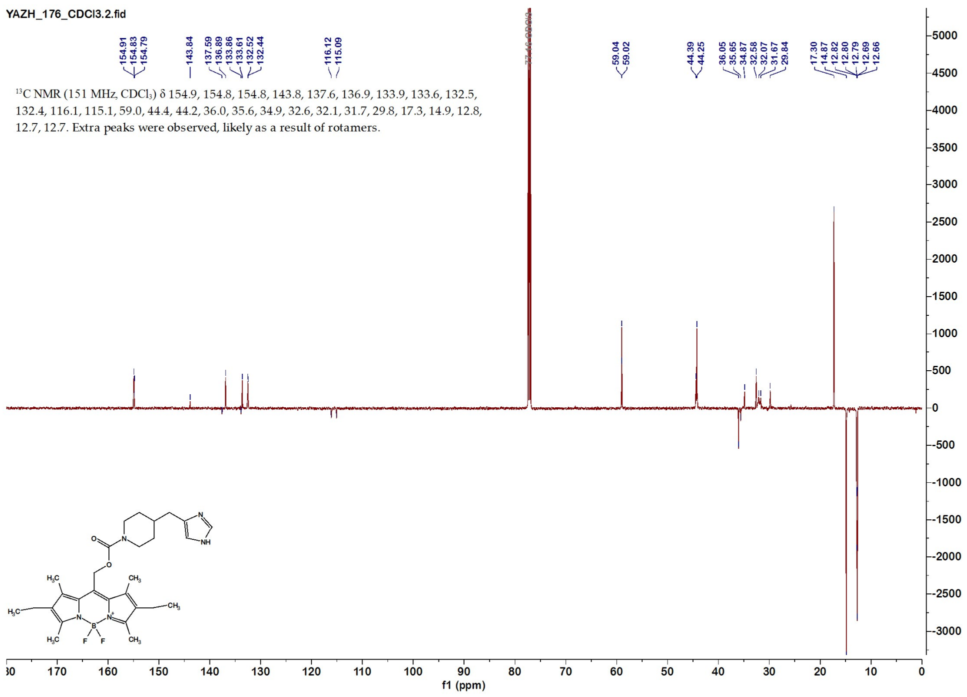

Figure S22. ^{13}C NMR spectrum of final compound **5**.

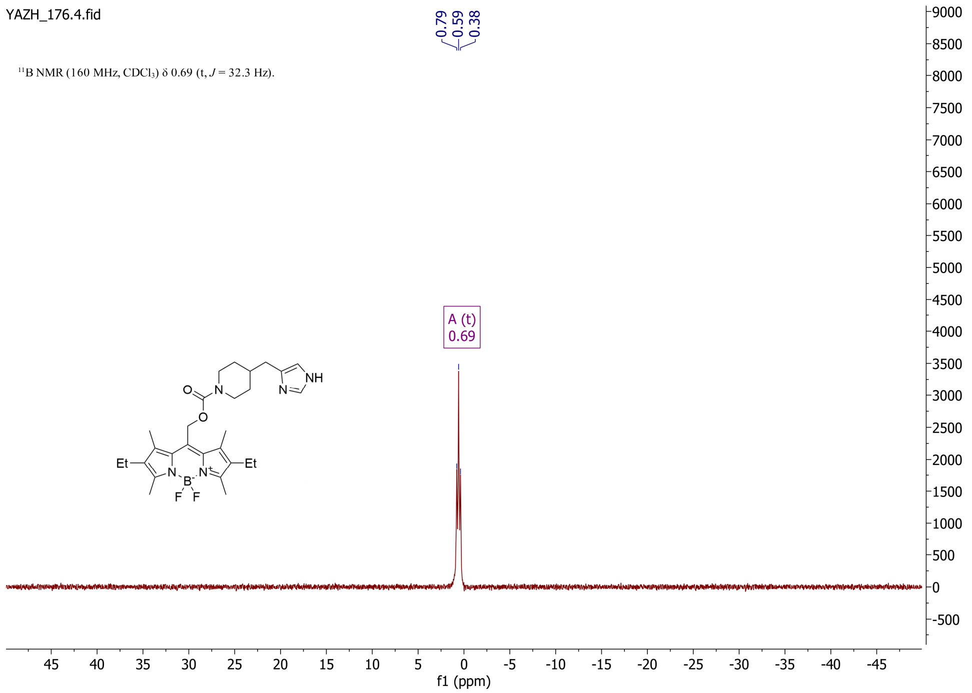

Figure S23. ^{11}B NMR spectrum of final compound **5**.

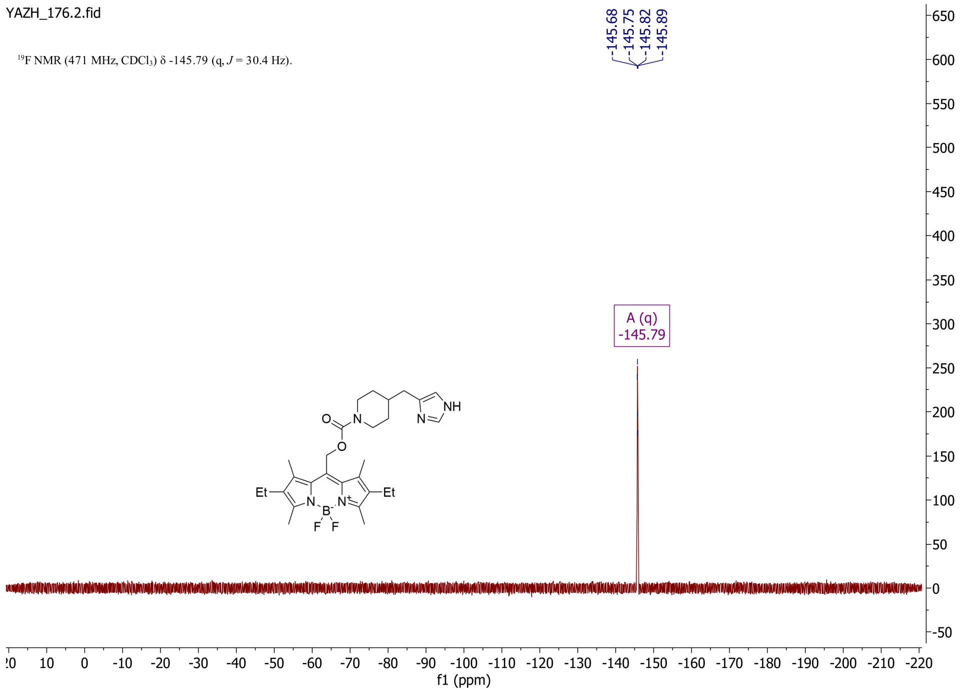

Figure S24. ^{19}F NMR spectrum of final compound **5**.

Mass Spectrum SmartFormula Report

Analysis Info

Analysis Name	C:\Documents and Settings\Administrator\Desktop\Hans\Week 23\20210607-Hans-formatetune_10_01_110.d	Acquisition Date	6/7/2021 3:56:23 PM
Method	servicems-100-500 (formate tune mix).m	Operator	demo
Sample Name	20210607-Hans-formatetune	Instrument / Ser#	micrOTOF-Q 10
Comment			

Acquisition Parameter

Source Type	ESI	Ion Polarity	Positive	Set Nebulizer	0.6 Bar
Focus	Not active	Set Capillary	4500 V	Set Dry Heater	200 °C
Scan Begin	50 m/z	Set End Plate Offset	-500 V	Set Dry Gas	8.0 l/min
Scan End	3000 m/z	Set Collision Cell RF	300.0 Vpp	Set Divert Valve	Source

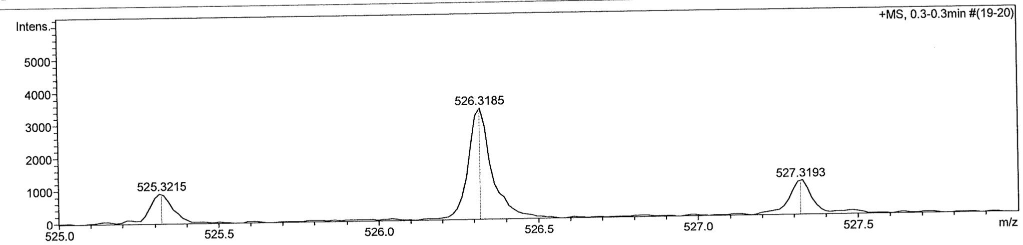

Meas. m/z	Formula	m/z	|err| [ppm]	err [mDa]	rdb	N-Rule	e⁻ Conf	mSigma	# Sigma
526.3185	C 28 H 39 B F 2 N 5 O 2	526.3164	3.8	-2.0	11.5	ok	even	30.7	1

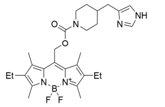

Bruker Compass DataAnalysis 4.0 printed: 6/9/2021 2:16:26 PM Page 1 of 1

Figure S25. HRMS spectrum of final compound **5**.

Acquired by	: Admin
Date Acquired	: 23/6/2021 10:17:40 AM
Sample Name	: YAZH_181_purified
Sample ID	:
Tray#	: 1
Vial#	: 5
Injection Volume	: 20
Data File	: C:\LabSolutions\Data\2021\2021-wk25\YAZH_181_purified.lcd
Background File	: blanco 23062021.lcd
Method File	: Method SCAN ACID standard.lcm
Report Format	: DefaultLCMS.lcr
Tuning File	: C:\LabSolutions\Tuning\tuning 15042021.lct
Processed by	: Admin
Modified Date	: 23/6/2021 12:16:07 PM

PDA Graph

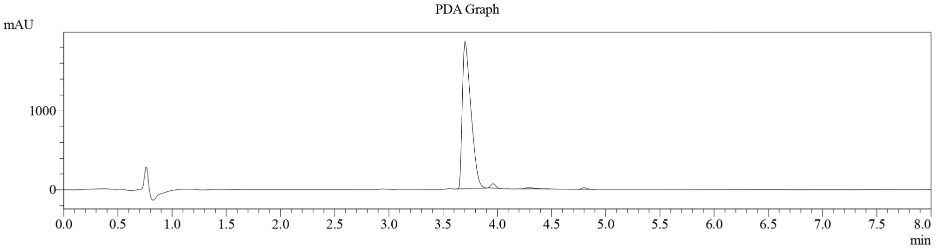

PeakTable

PDA Ch1 230nm 4nm

Peak#	Name	Ret. Time	Area	Area %
1		3.698	10273222	96.849
2		3.957	163161	1.538
3		4.284	101552	0.957
4		4.796	69532	0.655

MS Chromatogram
YAZH_181_purified C:\LabSolutions\Data\2021\2021-wk25\YAZH_181_purified.lcd

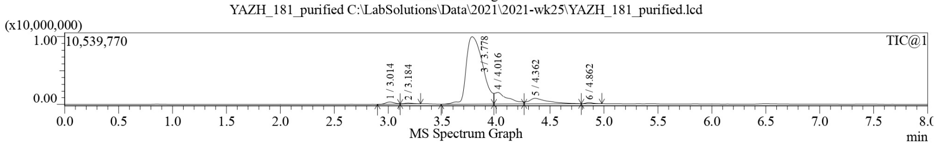

MS Spectrum Graph

#:1 Ret.Time:Averaged 3.770-3.790(Scan#:378-380)
BG Mode:Calc 3.490<->3.980(350<->399)
Mass Peaks:13 Base Peak:486.35(4171486) Polarity:Pos Segment1 - Event1

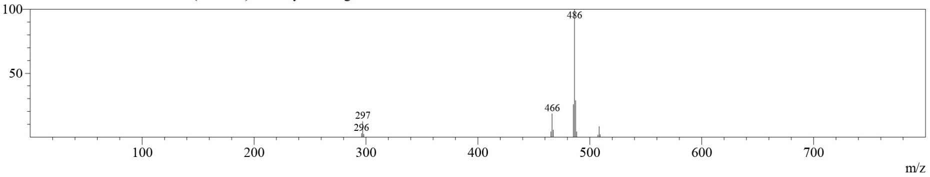

MS Spectrum Table

#:1 Ret.Time:
BG Mode:Calc 3.490<->3.980(350<->399)
Mass Peaks:13 Base Peak:486.35(4171486) Polarity:Pos Segment1 - Event1

#	m/z	Abs.Inten.	Rel.Inten.	Charge	Polarity	Monoisotopic
1	296.10	124613	2.99			
2	297.10	510932	12.25			
3	298.15	100905	2.42			
4	465.30	182931	4.39			
5	466.30	767534	18.40			
6	467.30	237233	5.69			
7	485.30	1065553	25.54			
8	486.35	4171486	100.00			
9	487.35	1196218	28.68			
10	488.35	182102	4.37			
11	507.30	77071	1.85			
12	508.35	347791	8.34			
13	509.30	90988	2.18			

Figure S26. LCMS chromatogram of final compound **6**.

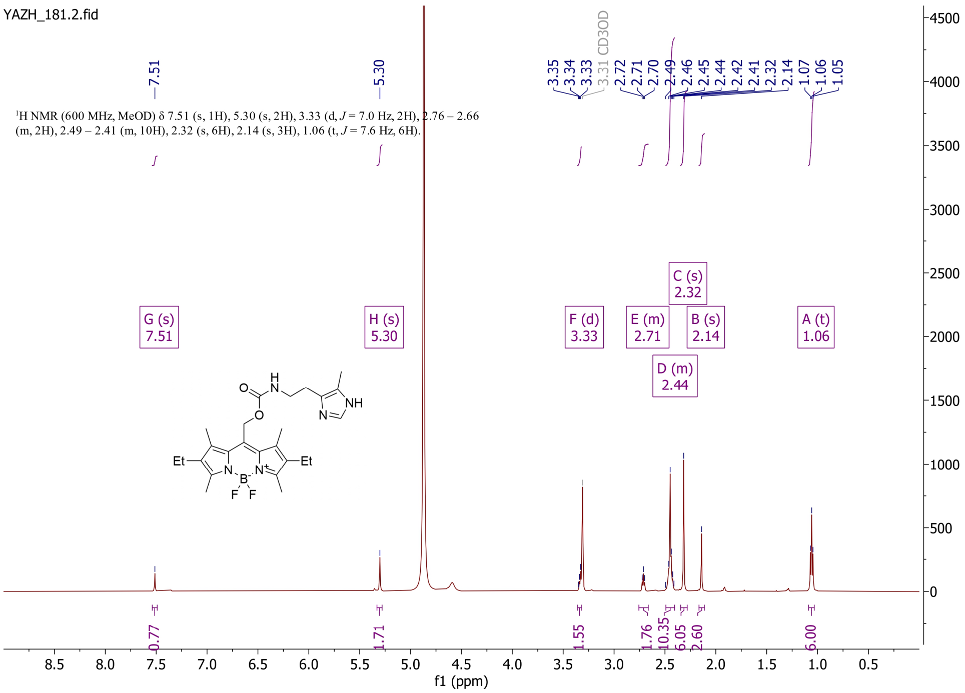

Figure S27. ^{1}H NMR spectrum of final compound **6**.

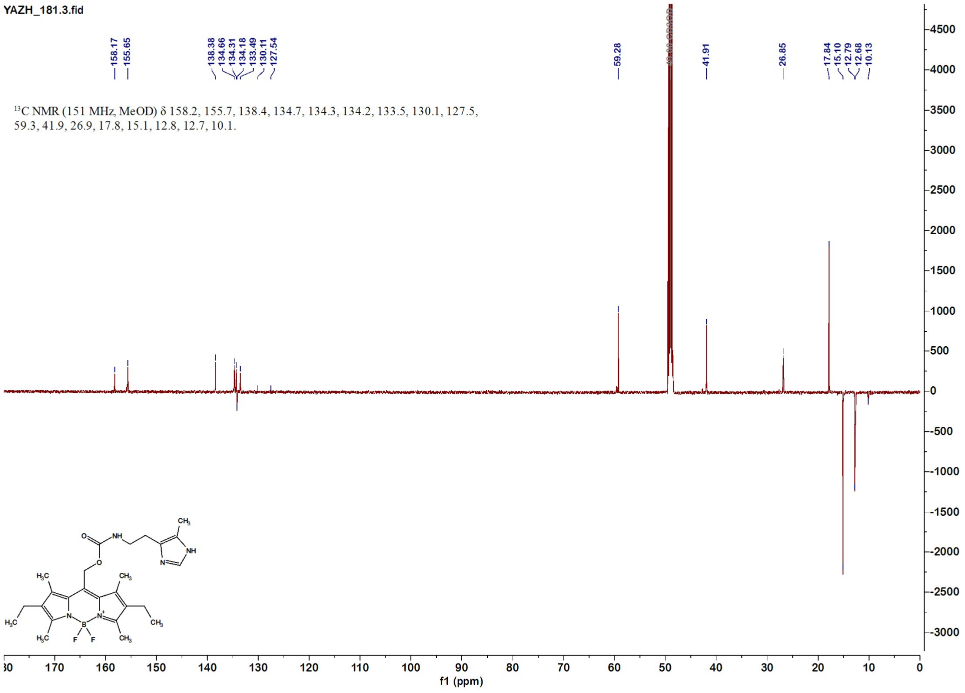

Figure S28. ^{13}C NMR spectrum of final compound **6**.

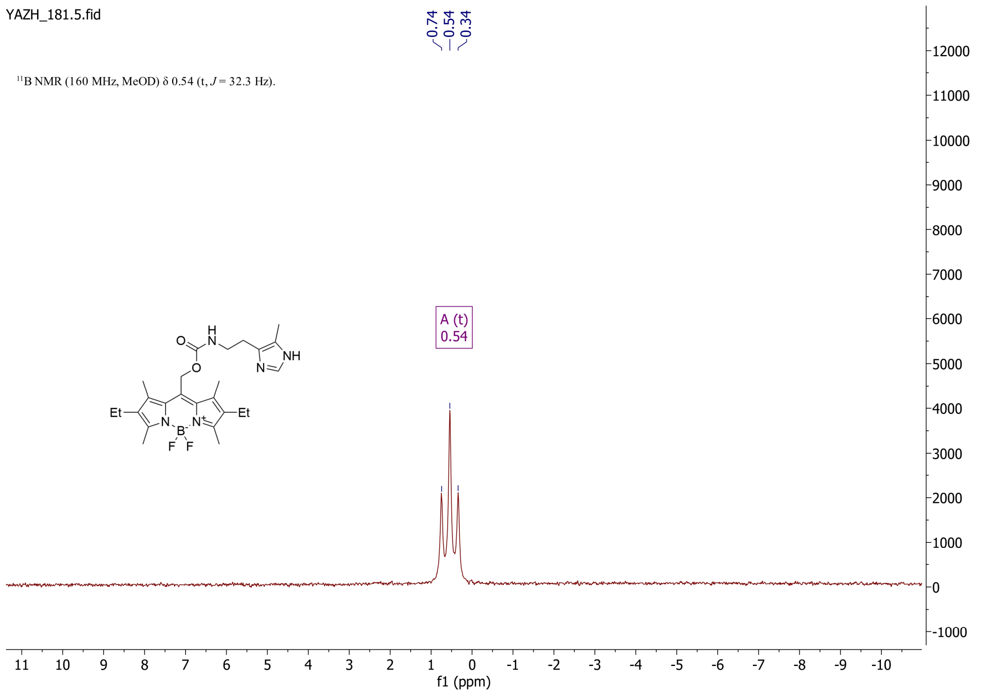

Figure S29. ^{11}B NMR spectrum of final compound **6**.

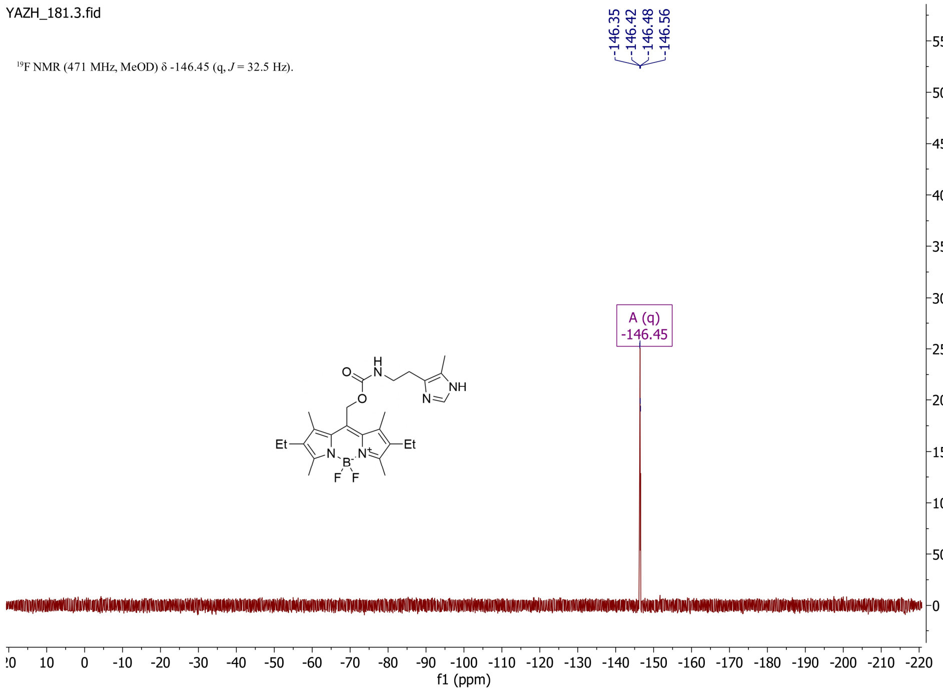

Figure S30. ^{19}F NMR spectrum of final compound **6**.

Mass Spectrum SmartFormula Report

Analysis Info

Analysis Name	C:\Documents and Settings\Administrator\Desktop\Hans\Week 23\20210607-Hans-formatetune_11_01_111.d	Acquisition Date	6/7/2021 4:00:21 PM
Method	servicems-100-500 (formate tune mix).m	Operator	demo
Sample Name	20210607-Hans-formatetune	Instrument / Ser#	micrOTOF-Q 10
Comment	YAZH-177		

Acquisition Parameter

Source Type	ESI	Ion Polarity	Positive	Set Nebulizer	0.6 Bar
Focus	Not active	Set Capillary	4500 V	Set Dry Heater	200 °C
Scan Begin	50 m/z	Set End Plate Offset	-500 V	Set Dry Gas	8.0 l/min
Scan End	3000 m/z	Set Collision Cell RF	300.0 Vpp	Set Divert Valve	Source

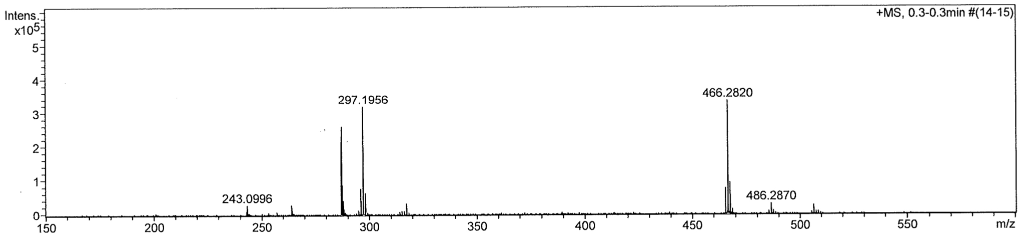

Meas. m/z	Formula	m/z	|err| [ppm]	err [mDa]	rdb	N-Rule	e⁻ Conf	mSigma	# Sigma
486.2870	C 25 H 35 B F 2 N 5 O 2	486.2851	3.8	-1.9	10.5	ok	even	38.1	1

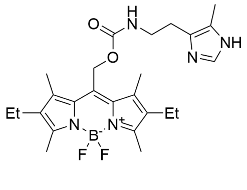

Bruker Compass DataAnalysis 4.0 printed: 6/9/2021 11:41:22 AM Page 1 of 1

Figure S31. HRMS spectrum of final compound **6**.
